# Money Affects Theory of Mind Differently by Gender

**DOI:** 10.1371/journal.pone.0143973

**Published:** 2015-12-03

**Authors:** Garret Ridinger, Michael McBride

**Affiliations:** Experimental Social Science Laboratory, Department of Economics, University of California Irvine, Irvine, California, United States of America; University of Groningen, NETHERLANDS

## Abstract

Theory of Mind (ToM) ─ the ability to understand other’s thoughts, intentions, and emotions ─ is important for navigating interpersonal relationships, avoiding conflict, and empathizing. Prior research has identified many factors that affect one’s ToM ability, but little work has examined how different kinds of monetary incentives affect ToM ability. We ask: Does money affect ToM ability? If so, how does the effect depend on the structure of monetary incentives? How do the differences depend on gender? We hypothesize that money will affect ToM ability differently by gender: monetary rewards increase males’ motivation to express ToM ability while simultaneously crowding out females’ motivation. This prediction is confirmed in an experiment that varies the structure of monetary rewards for correct answers in the Reading the Mind in the Eyes Test (RMET). RMET scores decrease for females and increase for males with individual payments, and this effect is stronger with competitively-structured payments. RMET scores do not significantly change when monetary earnings go to a charity. Whether money improves or hinders ToM ability, and, hence, success in social interactions, thus depends on the interaction of gender and monetary incentive structure.

## Introduction

A wide array of evidence finds that money affects social relationships. Studies by economists identify how money incentives action and facilitates trade of goods and services across communities and nations, thus serving as an effective tool for encouraging large-scale economic cooperation [1, 2]. However, psychologists' have shown that money can damage social relationships and lead individuals to pursue goals individually without aid from others, thus hindering success in some domains of life [3–6].

This study considers Theory of Mind (ToM) ─ the ability to assess and predict others' thoughts, intentions, emotions, and behaviors ─ as a mediating factor between money and behavior in social settings. We ask: Does the presence of money in an interpersonal interaction affect ToM ability? If so, how does the affect of money on ToM ability depend on the structure of the monetary incentives? Do the gender differences in motivation and crowding out found in other settings also reveal themselves with ToM ability? To what extent do the gender differences depend on the competitiveness of the setting?

Previous studies have found that ToM allows an individual to navigate interpersonal relationships, avoid destructive conflict, and empathize [7–10], and that impairment of one's TOM ability is associated with difficulty in maintaining positive relationships [11, 12]. ToM ability is especially important in highly strategic environments in which success depends on the ability to not only understand others' intentions but also predict their behavior [13–15]. Whereas humans beyond the age of 5 typically can be said to have ToM, it is also the case that the degree to which it is expressed, what is our primary interest and what we herein refer to as "ToM ability," can vary across persons and settings. For example, individuals higher on the autism spectrum manifest lower ToM ability [11, 12], females manifest higher ToM ability on average [16], and ToM ability can be enhanced by reading literary fiction [17].

Money could potentially affect ToM ability via multiple channels. Money is a powerful motivating force that could potentially create a large incentive to understand others' mental or emotional states. That money motivates, with more money inspiring more motivation, is a fundamental premise of experimental economics, so much so that experimental economists typically use monetary payments in their experiments to create a strong incentive to make decisions seriously [18]. Experimental psychologists agree that money provides strong extrinsic motivation but also find that money can "crowd out" other motivations that individuals might have [19–22] and make individuals more inward-focused [23–25]. These different findings suggest that the effect of money on an individual's ToM ability is unclear.

Gender is an additional complicating factor. While some studies have found contrary results [26,27], the majority of studies, confirmed by meta-analysis, show a female advantage in recognizing emotional cues, an important facet of ToM ability [16, 28–31]. Moreover, previous experiments have found crowding-out effects on motivation to be larger on average for females, who, relative to males, appear to have higher intrinsic motivation to manifest empathy [22, 32–35]. Competition is also found to affect motivation differently by gender; on average, men seek out competition at higher rates than women [36–38]. Other evidence suggests that competition itself can lead to differential effects based on gender. Research using meta-analyses have found a small but significant gender effect in negotiation performance [39, 40]. Additional studies have found gender differences in real effort tasks when subjects are paid via tournaments [38, 41, 42]. Indeed, these gender differences are believed to have large consequences for life outcomes. The large and persistent wage differentials between women and men have been attributed in part to women sorting into less-competitive career paths [43, 44] and less frequently asking for raises [45]. If ToM ability depends in part on motivation, then these gender differences in motivation found in prior literature may be found in the relationship between money and ToM ability. Such might be the case, for example, if monetary incentives differently affects males' and females' motivation to understand others' emotions. Our paper further explores this possibility.

Two studies have considered the effect of money on the ability to accurately interpret others thoughts or feelings, an important component of ToM ability. In Klein and Hodges [35], subjects watched videotaped recordings of other students (targets) discussing academic problems and reported what they thought the targets were feeling. They found that monetary incentives increased accuracy for both male and female subjects and eliminated the gender differences in accuracy found in the non-monetary control condition. Before giving their reports, subjects in the money condition were told, "It was important to us that you try your best at inferring the thoughts and feelings of this person, so we will reward your accurate performance with money." Subjects in the non-money condition were not told this. It is unknown whether this language provided the money-condition subjects with additional, possibly confounding, motivation to perform well because the experimenter explicitly requested that they do so.

Ma-Kellams and Blascovich [46] conducted a similar experiment but with different incentive schemes. In Klein and Hodges [35], the subject is told that each exactly correct answer receives $2 and each somewhat correct answer receives $1. In Ma-Kellams and Blascovich [46], the money-condition participants are told that they had the opportunity to earn a financial reward if they performed well, and those that achieved high performance (98%) would earn financial prize; the non-monetary participants were told that they had the opportunity to earn points and that the person who gained the most points winning a prize. The scheme for the non-money participants is inherently competitive, but the scheme for the money participants is not. It is unclear whether different perceptions of competition across the conditions acted as a confound. This paper also does not explore gender differences.

Our study differs from these prior studies in two important ways. First, we use the Reading the Mind in the Eyes Test (RMET) [12] to measure a subjects' ability to assess others' thoughts and emotions. The RMET task has been used by many researchers to study ToM ability [12, 17, 16, 25, 47–49], and we chose to use it because it has many features that are beneficial for our study. For one, prior studies have found that it correlates strongly with many factors believed to affect ToM ability. For example, other higher order theory of mind tests include the Strange Stories Test [50], Faux Pas Test [51, 52], Reading the Mind in the Voice Test [53], and the Cambridge Mindreading Face-Voice Battery Test [54]. Studies have found positive correlations between the RMET with the Faux Pas Test [55], Reading the Mind in the Voice Test [56], and the Cambridge Mindreading Face-Voice Battery Test [54]. However, other studies found that scores in the RMET were not correlated with the Strange Stories Test [57, 58] and the Faux Pas Test [52, 57]. Another nice feature of the RMET is that it generates a wide distribution of scores that is conducive to standard statistical procedures. We can also use third-party assessments to validate what the task considers to be correct answers. Second, we use a wider array of monetary incentive schemes than used in prior studies. Our experiment places subjects into different conditions that mimic different ways that monetary incentives might arise in social interactions. This design enables us to identify how different monetary incentives affect the ToM of males and females.

Drawing from different strands of experimental research on ToM ability and the impact of money on interpersonal relationships, we hypothesize that money in our experiment will affect ToM ability as measured by RMET differently by gender: monetary rewards increase males’ motivation to express ToM ability while simultaneously crowding out females’ motivation. This prediction is confirmed: RMET scores decrease for females and increase for males with individual payments, and this effect is stronger with competitively-structured payments. RMET scores do not significantly change when monetary earnings go to a charity. Whether money improves or hinders ToM ability, and, hence, success in social interactions, thus depends on the interaction of gender and monetary incentive structure.

## Theory of Mind and Gender

Given the prior literature mentioned above, we here provide a conceptual framework useful for understanding how money can affect ToM and in generating testable predictions. The ToM ability that an individual manifests in a setting can be represented by this simplified equation:

$$ToMability_{igs}=fixed_{ig}+engagement_{igs},$$

where *ToMability*_*igs*_ is the ToM expressed or realized by individual *i* of gender *g* in a particular setting *s*, *fixed*_*ig*_ is that part of an individual's ToM ability that is fixed across settings, and *engagement*_*igs*_ is the degree to which the individual is socially engaged to express ToM in a given setting. An individual high on the autism spectrum can be viewed as having a reduced fixed component of ToM ability [7, 8], while reading a passage of literary fiction temporarily increases one's engagement [12]. To be clear, this mathematical formulation is for conceptual purposes only. We do not mean to assert that these factors must be related in additive or linear fashion in actual individuals.

We conjecture that money affects one's engagement rather than innate ability and does so through three separate channels: intrinsic motivation, extrinsic motivation, and social orientation. A simple representation of engagement can be written as:

$$engagement_{igs}=intrinsic_{igs}+extrinsic_{igs}+orientation_{igs},$$

where *instrinsic*_*igs*_ and *extrinsic*_*igs*_ are the intrinsic and extrinsic motivation of an individual *i* of gender *g* in a given setting *s* and *orientation*_*igs*_ represents the degree to which the individual is outwardly or socially-oriented in the setting. Again, the additive formulation is merely to illustrate how the different components might individually impact engagement and is not meant to be a literal representation.

As stated earlier, prior studies establish that both intrinsic and extrinsic motivation are important components in an individual’s desire to engage in a given social situation [19, 32, 33, 36], and a monetary incentive may actually lower overall motivation in some settings if it decreases (i.e., "crowds out") intrinsic motivation more than it increases extrinsic motivation [19–22]. This crowding out effect is stronger for females [22, 34], who, relative to males, appear to have higher intrinsic motivation to manifest empathy [32, 33, 35]. As a result of motivational crowding out, females' overall ToM ability may actually decrease when given monetary incentives. Conversely, because intrinsic motivation in males is relatively low, their crowding out may be too small to offset the increase in extrinsic motivation. Moreover, given the evidence that men seek out competition and women avoid it [36–38], competitively-structured monetary rewards should have an additional positive effect on males' extrinsic motivation leading to an even stronger, positive effect on males' engagement and ToM ability. The effect of competition would be the opposite for females, further driving down their engagement and ToM ability.

Social orientation can be defined as the weight individuals place on their own and other’s welfare [59]. An inward orientation occurs when individuals who place relatively little weight on others’ welfare, whereas an outward orientation occurs when people place relatively more weight on others' welfare of others. The direction of one's social orientation—whether inward or outward—has been shown to affect behavior [23–25]. Merely priming subjects with the concept of money has been found to reduce the likelihood that they ask others for help and to reduce the degree in which they assist others who ask for help, effectively inducing an inward orientation [5, 6]. While money may prime an inward orientation, there is reason to suspect that participating in social activities like charity may prime an outward orientation. First, empirical studies on adolescents and young adults have shown that volunteering for charity can reduce the frequency of anti-social behaviors [60–62]. Second, an increase in volunteering and charitable giving is associated with a higher likelihood that a person has a pro-social orientation [59, 63–65] Important for us is that social orientation has been shown to be related to ToM ability; recent evidence finds higher ToM ability from individuals who are naturally more socially oriented or primed to be socially oriented [23–25]. These effects appear to be similar across gender, suggesting that the introduction of money may negatively affect TOM ability for both males and females by shifting orientation inward. Gender differences in the effect of money on ToM ability should thus largely arise from differential effects on motivation rather than orientation.

## Methods

### Conditions

We tested the above claims by placing subjects into one of four experimental conditions. Condition 1 (females = 41; males = 23) was our Baseline condition which replicates the (unincentivized) RMET task described above without any incentive provided by the researcher, as typically done in other studies. Upon arrival at the laboratory at the start time of the experiment, each subject was randomly assigned to one of the computer terminals. After advancing through multiple screens of instructions, the subject was shown a screen that contains a cropped photograph of the eyes of an individual and a list of four possible emotions. The subject was also asked on that screen to select which of the four emotions best matched the eyes in the image. A printout copy of definitions for each of the emotions was provided to the subject to reference throughout the experiment. The subject then used the mouse to select one of the four emotions. After making a selection, the screen advanced to the next pair of eyes with the accompanying different list of emotions from which to select. This procedure was repeated thirty-six times, with a different individual in each photograph and a different list of emotions for each corresponding photograph. Subjects were not told at the beginning how many photographs they would see, however, subjects were told during the recruitment phase that the experiment would not exceed 1.5 hours. Feedback on performance was not given until the end of the task, at which time the subject was told how many of the thirty-six she correctly answered and asked to complete a questionnaire. As part of the questionnaire, the subject undertook the Cognitive Reflection Test [66], which has been shown to be strongly correlated with other measures of intelligence [49]. S1 Appendix provides screenshots of the instructions, a screenshot of a sample RMET question as presented in the experiment software, the full set of RMET images, the list of definitions, and the correct answers.

Condition 2 (females = 40; males = 18) provided an Individual incentive. This condition was identical to the Baseline except the subject was paid $0.40 for each correct selection in the RMET. The instructions in this treatment only differed from the baseline by the addition of one sentence that read: "For each correct choice you will receive $0.40." This text constituted a minimally-primed monetary incentive as no other attempt was made to prime the notion of money. Payment earnings were distributed privately one at a time to each subject at the end of the experiment session.

Condition 3 (females = 37; males = 27) was the Winner-take-all condition. This condition was identical to the Baseline except the subjects were randomly and anonymously placed into groups of four via the computer and then told that the subject within the group that performs best on the RMET will receive $40 and all others in that group receive $0 (a random draw determines winner in case of tie). The $40 was chosen to roughly equalize the monetary earnings across conditions 2–4; average earnings were approximately $10 per person in the Individual condition, thus making a prize of $40 akin to the winner receiving the earnings for everyone in the group.

Condition 4 (females = 26; males = 26) was the Charity condition. Before doing the RMET, the subject was told that he or she would undertake a task for a charity of her choice, with the amount donated anonymously on the subject's behalf to the charity based on his or her performance on the task. The subject was then given a list of four charities (Amnesty International, UNICEF, Doctors without Borders, and American Cancer Society) and provided with a paragraph about that organization's mission and a picture of an example of a beneficiary of that organization. The text and pictures were meant to serve two purposes: to enable the subject to make an informed choice when deciding the charity to receive the earnings, and to prime an outward, other-regarding orientation. The subject next selected which charity will receive his or her earnings and then completed the RMET with $0.40 per correct question donated to the selected charity. All other aspects of this condition were the same as in the Baseline. Payment earnings were distributed to the selected charities after the completion of the experiment.

Table 1 provides a summary of our predictions for each condition relative to the Baseline. The '+' and '-' in the table indicate our predicted directional average effect, with more symbols indicating a larger effect. An unclear average effect is indicated by '+/-'. The monetary incentive in the Individual condition should: decrease intrinsic motivation on average for females but have little to no effect on males on average (column A); increase both males' and females' extrinsic motivation (column B), with the effect larger for males; and reduce social orientation leading individuals to be more self-oriented (column C). Aggregating these channels, the Individual condition will have an overall, average, negative effect for females and an overall, average, positive effect for males (column D). We predict similar effects in the Winner-take-all condition, albeit stronger due to the different responses to competition. In the Charity condition, the negative impact of money on orientation should be offset by the outward orientation of doing the RMET task for others. The overall effect on orientation is unclear with no difference by gender. Gender differences should still be observed in motivation, so we predict no difference in females' RMET score in Charity relative to Baseline, but males may express slightly higher ToM. We emphasize that disaggregating by gender is vital: the apparently minimal changes with males and females combined (column E) mask large and significant gender differences (column D).

**Table 1:** Predicted average treatment effect on RMET score relative to baseline condition.

		(A)	(B)	(C)	(D)	(E)
		Intrinsic motivation	Extrinsic motivation	Social orientation	Overall gender-specific effect	Overall combined effect
(1)	Female	--	+	-	--	
Individual	Male	0	++	-	+	-
(2)	Female	--	+/-	-	---	
Winner-take-all	Male	0	++	-	++	-
(3)	Female	0	+	+/-	+/-	
Charity	Male	0	+	+/-	+	+

Columns A-C separate the different channels by which money is predicted to affect overall engagement. Symbols indicate the direction and magnitude direction of predicted effect: "++" indicates large positive effect, "+" indicates small positive effect, "0" indicates little to no effect, "+/-" indicates an unclear or no effect, "-" indicates a small negative effect, "--" indicates a large negative, and "---" indicates a very large negative effect. Gender-specific treatment effects are predicted in motivation but not orientation. Large gender differences in the Individual and Winner-take-all conditions (column D) are obscured when males and females are combined (column E).

### Participants

A total of 238 students participated in our experiment that took place in a computer laboratory. Subjects were recruited from a laboratory subject pool that includes university students from the student body of the entire campus. The students in both the subject pool and our sample have diverse ethnic backgrounds and come from many different majors (see Table A in S1 Appendix). Prior to each experimental session, a number of students from the subject pool are randomly selected to receive an email informing them of the upcoming experiment. Students that receive that email then register for the experiment via the subject pool website. There were no exclusion restrictions other than that the subject must be 18 years of age or older, must be currently enrolled as student at the university, and cannot participate in more than one experiment session. All subjects received a show-up payment of $7, plus additional earnings based on their choices and the treatment condition. They are not given course credit. (All data are available for download in S1 Dataset and S2 Dataset. See S1 Readme for variable descriptions.)

### Ethical Considerations

This project was approved by the University of California─Irvine Institutional Review Board under protocol HS#2011–8378. Individuals provided informed consent via a four-step process. First, to enlist in the subject pool, individuals must read a consent document and then provide consent to register by clicking on a box on the registration page. This acknowledgement is recorded electronically. Second, once in the subject pool, the individual receives email notifications that provide information about location and expected duration of upcoming experiment sessions. Third, upon receipt of an email notification, the individual consents to participate by signing-up for a particular session by clicking on a link in the email. Finally, upon arrival at the laboratory for the experiment, the subject is verbally reminded that participation is voluntary, that she is free to go at any time without penalty, and that continuing to participate indicates that she has given consent to participate. This consent process was approved by the Institutional Review Board. Written consent was waived because this process was deemed sufficient to obtain informed consent.

## Results

**Fig 1:**
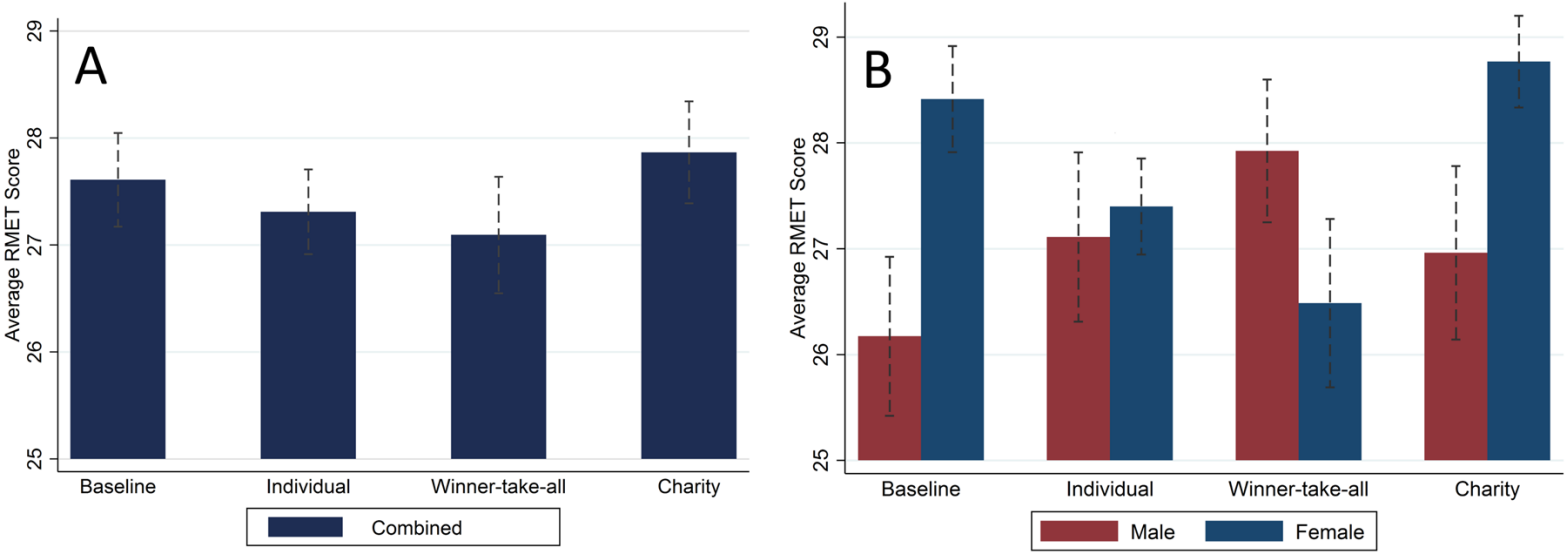
Unadjusted average RMET score by treatment. (A) Plots the average RMET score with males and females combined. (B) plots the average RMET score by gender. Dotted lines represent 95% confidence intervals. Combined averages move in the directions predicted in Table 1 but do not significantly differ across conditions. Gender-specific averages manifest much larger, often statistically significant, differences across conditions.

**Fig 2:**
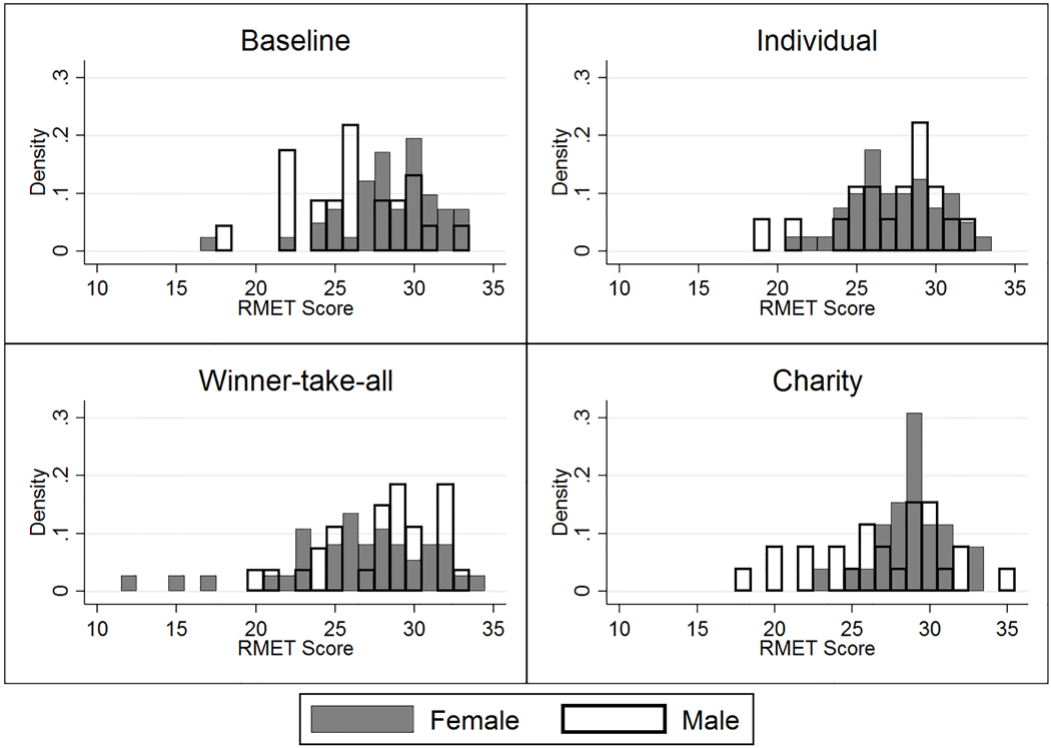
Histogram of unadjusted RMET scores by treatment. For a given RMET score, taller bars indicate a larger density of individuals with that score. Female and male distributions are represented with shaded bars and empty bars, respectively.

As seen in Fig 1A, there are only small differences in average RMET scores across the treatments when pooling males and females, and these differences are not statistically meaningful (see S1 Appendix). This finding could be used as *prima facie* evidence that money does not affect ToM ability, however, these combined averages mask significant gender differences revealed in Fig 1B that align with the predictions from Table 1. Females outscore males on the RMET on average by a statistically significant amount in the Baseline and Charity conditions, but do worse than males in the Winner-take-all condition. RMET scores are similar in the Individual condition. Fig 2 provides additional evidence that the effect of the treatment conditions differs by gender. The distribution of females' RMET scores shifts downward, while the distribution of males' RMET scores shifts upwards as we move from the Baseline to the Individual and Winner-take-all conditions. The variance in scores is similar across genders in the Baseline and Individual conditions, but the females' variance is larger in the Winner-take-all and smaller in the Charity conditions.

These figures provide some cursory evidence in support of some of our predictions. For example, as seen in Fig 2, the distribution of females' RMET scores is higher than that of males in the Baseline condition, but the reverse appears true in the Winner-take-all condition. However, these figures only provide imprecise substantiation in part because they do not account for other subject-level characteristics found in prior studies to affect RMET scores [16, 23, 47–49]. To obtain sharper estimates of the treatment effects, we conduct regression analyses with a number of controls. A gender dummy variable captures an average gender effect that persists across conditions. The average time taken by a subject to answer all RMET questions controls for subject-specific time spent on questions, potentially capturing difference in cognitive effort or other ability in completing the RMET. Whether English is the subject's first language and the number of years the subject has lived in the U.S. both capture the effect of different cultural backgrounds. Score on the Cognitive Reflection Test [66] provides a control of cognitive ability. Scores on the Cognitive Reflection Test were calculated as the sum of the correct answers to three questions. The Cronbach alpha for the three questions was 0.70 suggesting acceptable internal consistency. Controlling for these characteristics is particularly important as our sample is not perfectly balanced in these characteristics. The last four of these are not of primary interest to us and so are listed as "Other controls" in Table 2. We also calculate standard errors clustered at the subject level. As found in prior studies, being female, having English as the first language, spending more years in the U.S., and cognitive ability are all positively correlated with higher RMET score and statistically significant at standard confidence levels (typically p values less than 0.01).

**Table 2:** Ordinary least squares and random-effects probit regressions.

	Ordinary least-squares coefficients		Random-effects probit predicted changes in answering correctly	
Dependent variable	RMET score		Correct answer	
	(A)	(B)	(C)	(D)
Individual	-0.41		0.02	
	(0.57)		(0.02)	
Winner-take-all	-0.11		-0.02	
	(0.61)		(0.02)	
Charity	0.68		-0.00	
	(0.59)		(0.02)	
Individual x female		-1.42**		-0.04**
		(0.66)		(0.02)
Winner-take-all x female		-1.62**		-0.04**
		(0.82)		(0.02)
Charity x female		0.33		0.01
		(0.66)		(0.02)
Individual x male		0.95		0.03
		(1.04)		(0.03)
Winner-take-all x male		2.03**		0.06**
		(0.91)		(0.02)
Charity x male		1.48		0.04*
		(0.95)		(0.02)
Female	0.99**	2.87***	0.03**	0.03**
	(0.45)	(0.83)	(0.01)	(0.01)
Other controls	Yes***	Yes***	Yes***	Yes***
Subject specific effects	N/A	N/A	Yes	Yes
Question fixed effects	N/A	N/A	Yes	Yes
*N*	238	238	8568	8568
*R* ^2^	0.197	0.248		
*ρ*			0.04	0.04
*χ* ^2^			745.21	754.90

Columns A and B report results from ordinary least-squares regressions on subjects overall RMET score. Clustered standard errors at the subject level are reported in parentheses. Columns C and D report the change in predicted probability that a subject gives a correct answer in the RMET using random effects probit regressions that include subject random effects and question fixed effects. Standard errors are calculated using the delta method and are reported in parentheses. Significance is denoted as

* *p* < 0.10,

** *p* < 0.05,

*** *p* < 0.01.

See the Supporting Information for the probit regression coefficients from which estimates in columns 3–4 are calculated. Controls refer to variables Native English Speaker, Average Question Time, Cognitive Reflection Test, and Number of Years Lived in the U.S. The significance reported for the controls implies that we reject a test that these Controls are jointly equal to 0.

When not separating by gender, our ordinary least-squares estimates find little-to-no difference in overall average RMET scores across sessions (Table 2 column A). Consistent with our predictions, however, we find different effects of money on ToM ability for the different genders (column B). Relative to the Baseline, the Individual monetary incentive has a positive but statistically insignificant effect on males' RMET scores (β = 0.95, p = 0.36, 95% CI = -1.10 to 3.00), but a statistically meaningful negative effect on females' RMET scores (β = -1.42, p = 0.03, 95% CI = -2.72 to -0.12). Females' RMET scores are on average higher than males’ scores by a large and robust 2.9 (β = 2.87, p <0.01, 95% CI = 1.24 to 4.50). The crowding-out effect of the monetary incentive reduces overall engagement, but not enough to eliminate the females' overall advantage. Despite this, the male and female RMET scores in the Individual condition are not statistically different from each other (F-test, F(1,226) = 0.28, p = 0.60).

The competitive incentive significantly reduces RMET scores for females by about 1.6 (β = -1.62, p = 0.05, 95% CI = -3.22 to -0.12) and increases males' average RMET scores by about 2.0 (β = 2.03, p = 0.03, 95% CI = 0.24 to 3.8). While this change is large enough for men to perform better on average than women in the Winner-take-all setting despite the general female advantage, the difference is not statistically significant (F-test, F(1,226) = 0.73, p = 0.39).

The Charity condition has a positive but statistically insignificant effect on RMET scores for males (β = 1.48, p = 0.12, 95% CI = -0.40 to 3.36) and females (β = 0.33, p = 0.62, 95% CI = -0.98 to 1.64). Average female scores on the RMET are higher than males in the Charity condition (F-test, F(1,226) = 4.44, p = 0.04). Even if the very presence of money primes an inward orientation as found in other studies [5, 6], having the money donated to charity appears to prime a counteracting outward orientation. The former works to hinder ToM, while the latter enhances it, with a small net effect.

Altogether, although women have a fixed advantage of about 2.87 questions across all treatments when using all controls, whether this advantage implies higher average RMET scores depends on the treatment. For example, in the Winner-take-all, women do 1.62 worse and men 2.03 better, all else equal, for a 3.28 swing that has men outperform women on average on the RMET in the Winner-take-all condition by about 0.41 questions. Hence, men are outperforming women on average in the RMET in the Winner-take-all, though the difference is only 0.41 questions.

We assess the robustness of our results in various ways. First, we conducted additional regression analysis. Estimating random-effects probit regressions at the level of the question enables us to leverage the longitudinal data structure to control for individual subject and question effects. Estimated changes in the probability of getting an answer correct, as derived from the regressions, are reported in columns C-D of Table 2. The results are similar to the ordinary least-squares estimates except that the change in predicted probability for males in the Charity condition relative to the Baseline is now significant at the 10% level (p = 0.08, 95% CI = -0.01 to 0.09). Multiplying these predicted probability changes by 36 gives very similar predicted changes in overall RMET score similar to the OLS estimates. Additional regressions that vary control variables and assumptions about the standard errors were also estimated. Again, the estimates and their interpretation do not meaningfully change. Second, we checked if answers to specific RMET questions varied systematically across the conditions. They did not; the correct RMET answer was the modal selection by the subjects, the single exception being one question in the Baseline. It is the general ability to read emotions that appears to be affected by the monetary incentives. Finally, the average amount of time spent by the subjects in answering questions was the same across the Baseline, Individual, and Winner-take-all conditions but was slightly higher in the Charity condition. This difference in the Charity condition was solely due to women taking longer in that Condition. Again, it appears to be a general ability to read emotions that is affected by the incentives, an ability that is generally one that is not mediated through the amount of time spent.

## Discussion

Scholars have long distinguished between impersonal trade in large markets that is facilitated by money from the small-scale and interpersonal interactions between family members, friends, and neighbors that depend more on social preferences and norms rather than money [67–69]. We suspect that ToM ability is less important in the former, and thus any negative effect of money on ToM ability has a relatively small impact on the functioning of large-scale markets. ToM ability is, however, extremely important in small-scale economic and personal interactions. Experimental evidence has consistently shown that emotions are significant in explaining behavior in a number of situations including bargaining [70], pricing decisions [71], tax evasion [72], and charitable giving [73]. The ability to recognize the emotions of others may have a profound effect on individual decision making [74]. For example, in wage bargaining an employer with high ToM ability may be able to better anticipate how an offer may affect the emotions of their employee, and this anticipation may reduce potential conflict leading to better negotiation outcomes. Our study shows that the effect of money on ToM ability will depend in part on the structure of the monetary incentives in such settings. As our Charity condition reveals, it is not the presence of money per se but how monetary incentives are structured that matters for ToM ability.

Our study also provides new insights into gender differences in behavior and life outcomes. For example, wage differentials between women and men have been attributed in part to women less frequently asking for raises [43], and sorting into less-competitive career paths [43, 44]. When bargaining face to face, one experiment found that women were more likely to accept lower offers relative to men [75]. Women may be less likely to enter wage negotiations and more likely to accept unfair offers because they may recognize, as found in our study, that their ToM ability is relatively inhibited in that setting. This behavioral response to hindered ToM ability may contribute to lower wages for women. However, when women negotiate salaries for others, they seek higher compensation compared to when they negotiate for themselves [45, 76], a finding that is consistent with our findings as women’s TOM ability was not reduced in the Charity condition. For career choice, the type of occupation can differ greatly on the level of competiveness. One possibility is that women may avoid competitive settings in part because they assess that their ToM ability is impaired in such environments. Conversely, men may feel enhanced ToM ability in such environments and thus seek them out. The differential effect of money on ToM ability may thus contribute to gender differences in wages and career choice.

We also note a methodological implication of our study for future research on ToM ability. That women generally score higher on some ToM measures such as the RMET is partly a function of the common practice of not incentivizing the RMET task with money. Males can perform similarly to women on the RMET, despite the females' general average advantage, under some incentive schemes. Whether future researchers should or should not use monetary incentives when measuring ToM ability thus depends on the type of setting being studied. ToM ability in competitive environments, for example, may be more appropriately studied using an incentivized measure of ToM ability. Failing to account for these setting-specific factors can lead to inaccurate conclusions about gender-specific abilities in those settings, particularly when samples are gender-imbalanced. Differences in gender imbalance might help to explain, for example, why the findings of one money-effect study were not successfully replicated [77, 78].

Although our study has found gender differences in how monetary incentives affect ToM ability, we acknowledge that our study cannot identify the source of those gender differences found. Whether the differences are due to genes, culture, or a combination of both is unclear. A more appropriately-designed research design will be necessary to identify the biological and cultural factors behind the gender differences in ToM ability that we report.

## Supporting Information

S1 Appendix (PDF)

S1 Dataset (XLS)

S2 Dataset (DTA)

S1 Readme (PDF)
