# Supplementary material for: Money Affects Theory of Mind Differently by Gender
Source: PLoS One. 2015 Dec 3;10(12):e0143973. doi: 10.1371/journal.pone.0143973 (PMC4669079; doi:10.1371/journal.pone.0143973)
Supplement: S1 Appendix — (PDF) [file pone.0143973.s001.pdf]

Appendix: Money Affects Theory of Mind Differently by Gender*

Garret Ridinger[1] and Michael McBride[1,+]

[1]**Affiliations**: Experimental Social Science Laboratory, Department of Economics, University of California, Irvine, CA, 92697-5100

[+]Correspondence to: mcbride@uci.edu

1 Introduction

We here provide additional details of the results presented in the main article. We first describe the experimental design and show screenshots of the instructions viewed by a typical subject in each condition. Next we present descriptive statistics and hypothesis tests of the experiment data. Finally, we present additional regression analysis and robustness checks of our results.

2 Experimental Design

Each subject participated in one of four experimental conditions. At the start of the experiment, subjects viewed instructions and then completed the Reading the Mind in the

*__Acknowledgments__: Data are available from the authors upon request. Financial support for this study was provided by Army Research Office Award No. W911NF-11-1-0332 and Air Force Office of Scientific Research Award No. FA9550-10-1-0569. We are grateful to colleagues for their comments and suggestions, and to the Experimental Social Science Laboratory for the use of its facilities.

Eyes Test (RMET) [12]. In the Baseline condition, subjects viewed the standard instructions for the RMET and received no monetary payment based on their RMET performance. In the Individual condition, the instructions were the same as the Baseline condition except that individuals receive \$0.40 for each correct selection in the RMET. In the Competition condition, subjects were randomly divided into groups of four. Subjects were told that the person in their group that preforms best would receive \$40 and all other subjects would receive \$0. In the Charity condition, prior to the RMET task, the subject is told that he or she would undertake a task for a charity of his or her choice, with the amount donated anonymously on the subject's behalf to the charity based on his or her performance on the task. The subject is then given a list of four charities (Amnesty International, UNICEF, Doctors without Borders, and American Cancer Society), provided with a paragraph about that organization's mission and a picture of an example of a beneficiary of that organization. The subject next selects which charity will receive the earnings, and then does the RMET with \$0.40 per correct question donated to their selected charity. At the end of the experiment subjects then completed a questionnaire that included demographic questions and the Cognitive Reflection Test [66].

3 Experiment Screenshots

The experiment was programmed and conducted with the software z-Tree [79]. The following screenshots present the instructions viewed by a given subject in the experiment. We present examples of the instructions for each condition. All instructions were delivered to subjects via their computer screen.

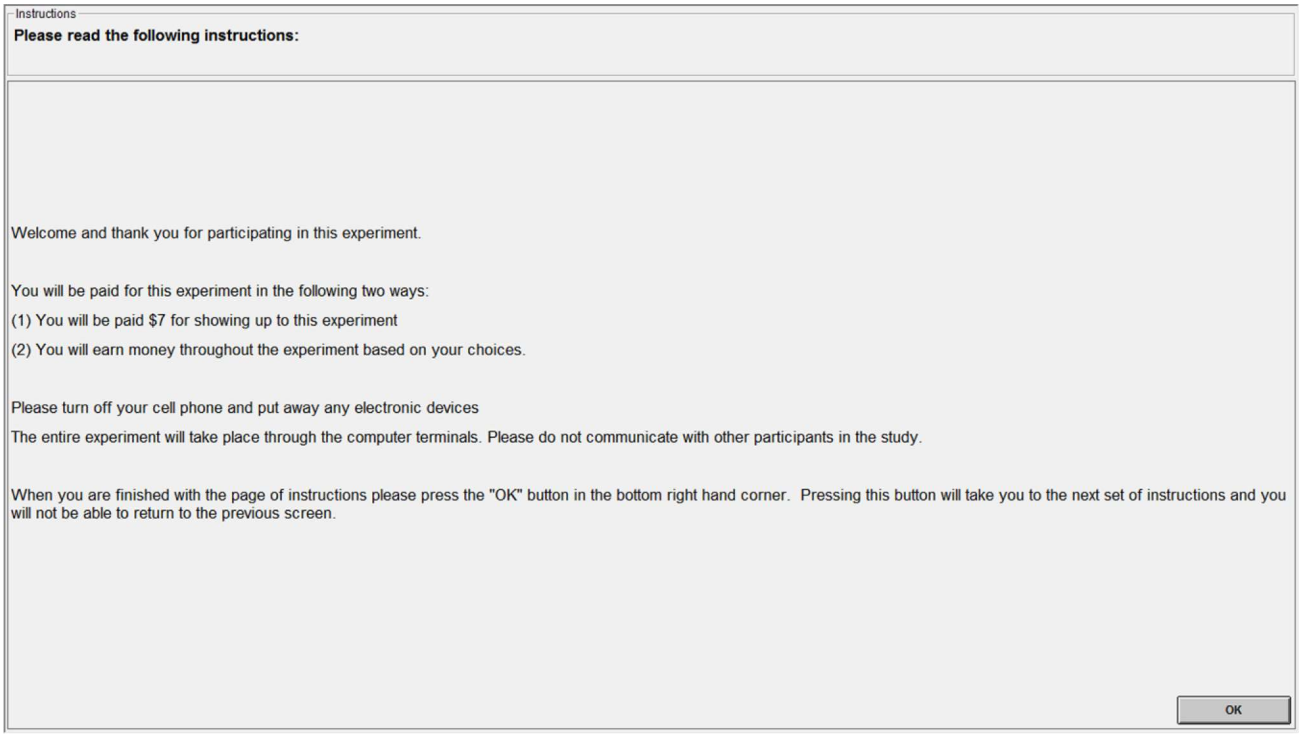

Fig A: Experiment Instructions—Welcome Screen for all Conditions

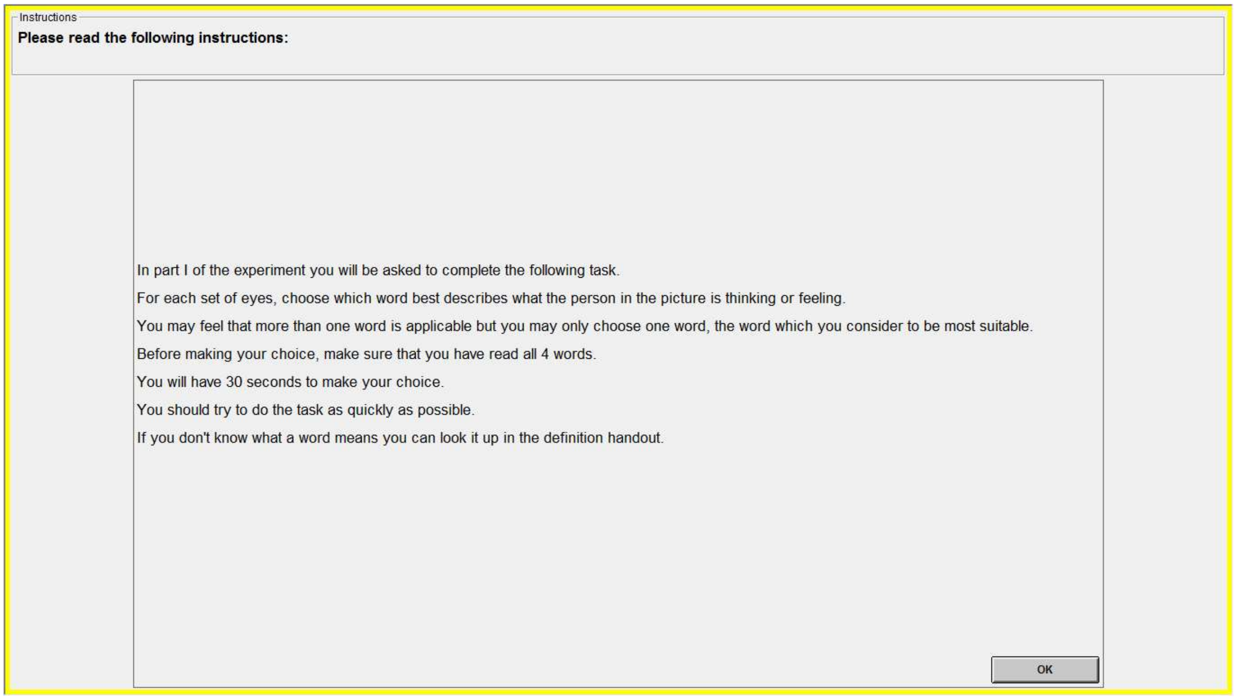

Fig B: Experiment Instructions—Baseline Condition

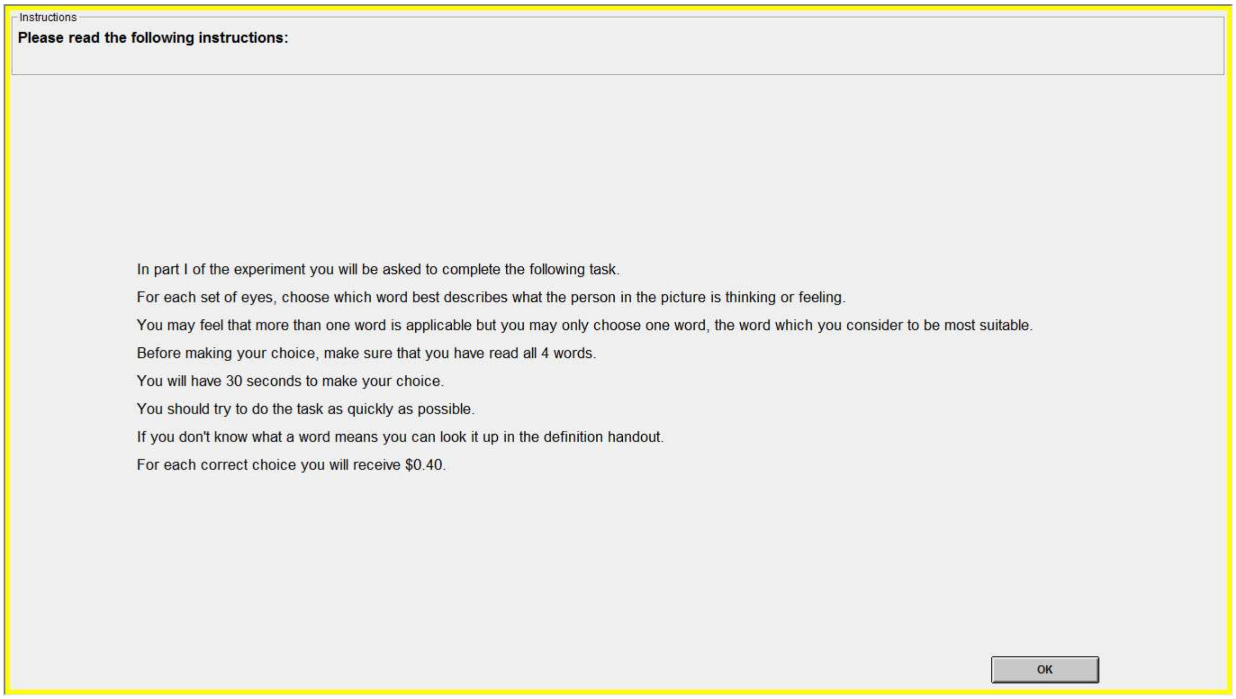

Fig C: Experiment Instructions—Individual Condition

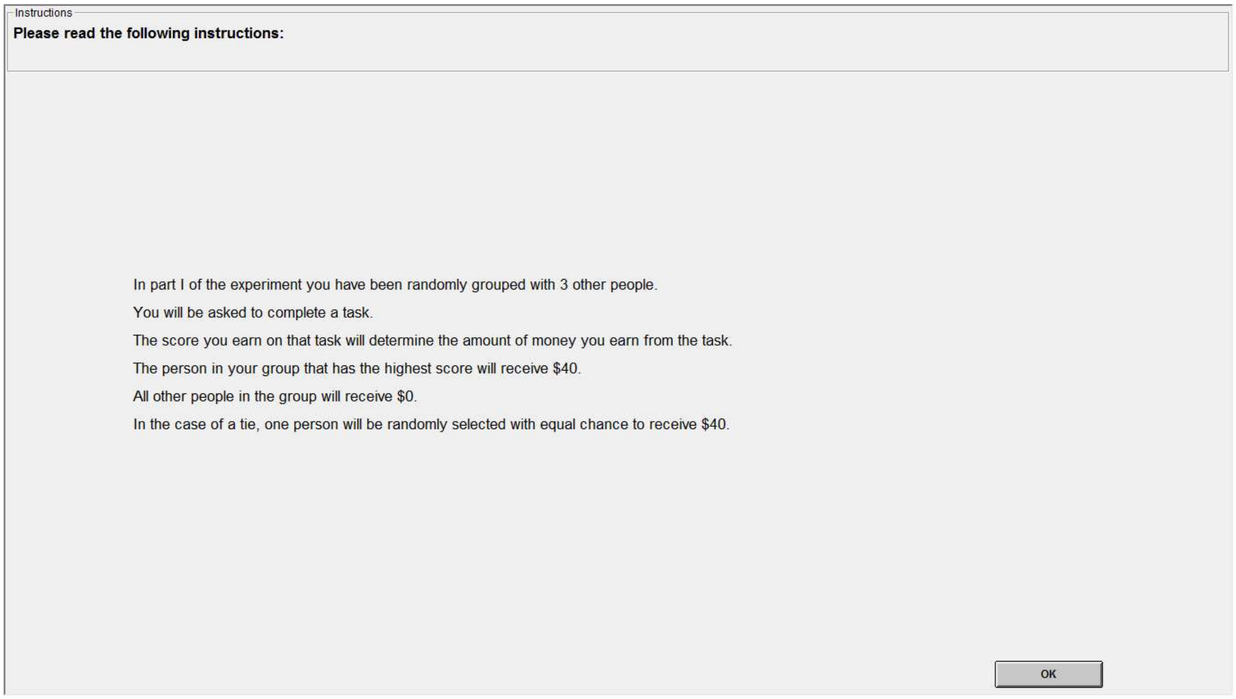

Fig D: Experiment Instructions—Winner-take-all Condition

Instructions

Please read the following instructions:

In part I of the experiment you will be asked to complete the following task.

For each set of eyes, choose which word best describes what the person in the picture is thinking or feeling.

You may feel that more than one word is applicable but you may only choose one word, the word which you consider to be most suitable.

Before making your choice, make sure that you have read all 4 words.

You will have 30 seconds to make your choice.

You should try to do the task as quickly as possible.

If you don't know what a word means you can look it up in the definition handout.

For each correct choice, one point will be added to your score.

The person in your group that has the highest score will receive $40.

All other people in the group will receive $0.

OK

Fig E: Experiment Instructions—Winner-take-all Condition, Continued

Instructions

Please read the following instructions:

In part I of the experiment you will be asked to complete a task for a charity.

The amount of money donated to the charity will be based on your performance in the task.

Prior to the start of the task, you will be asked to select one of four charities you would like the money to be donated to.

Please press OK to view descriptions of the four charities.

OK

Fig F: Experiment Instructions—Charity Condition

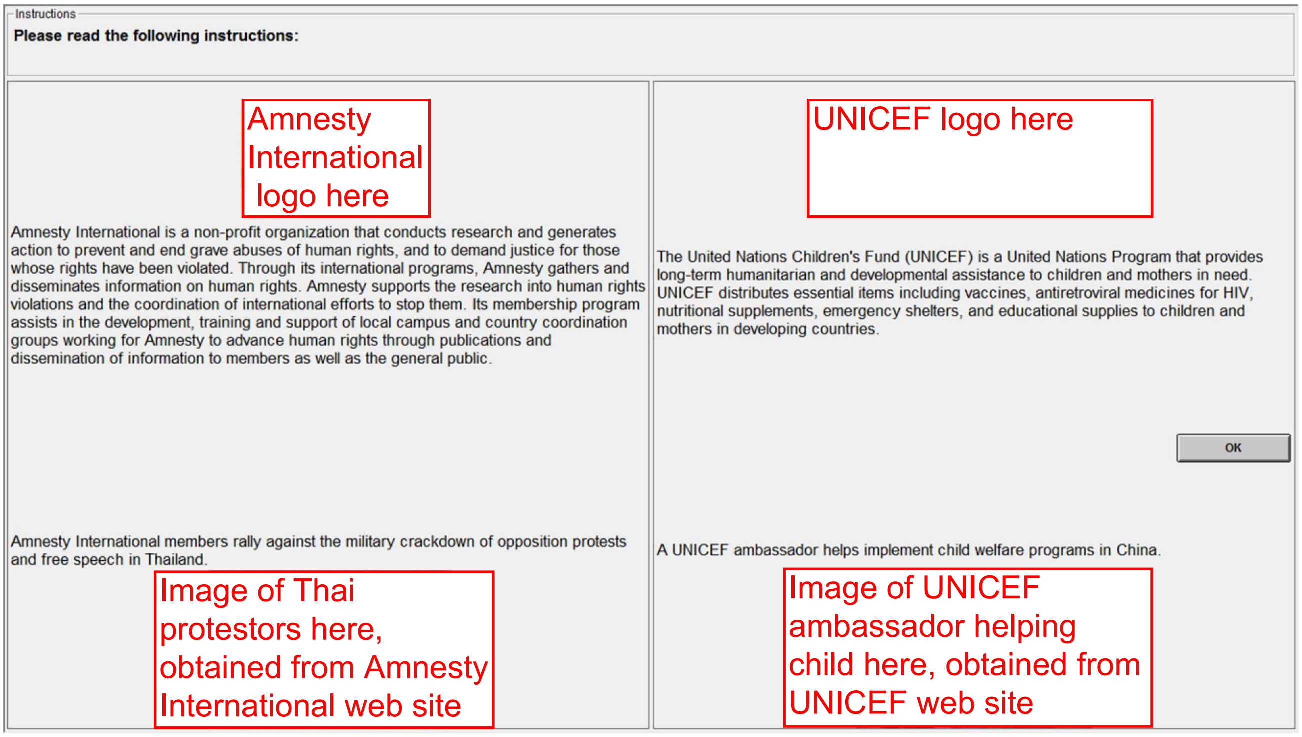

Fig G: Experiment Instructions—Charities I

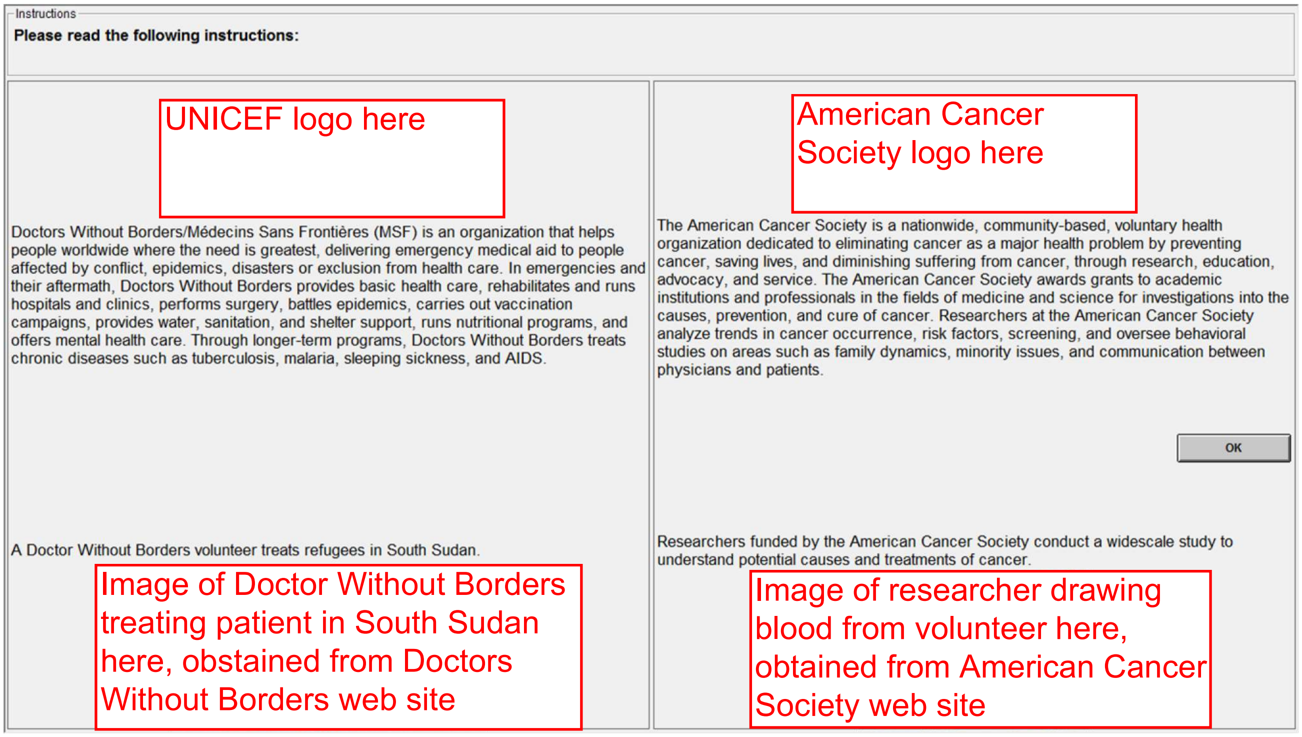

Fig H: Experiment Instructions—Charities II

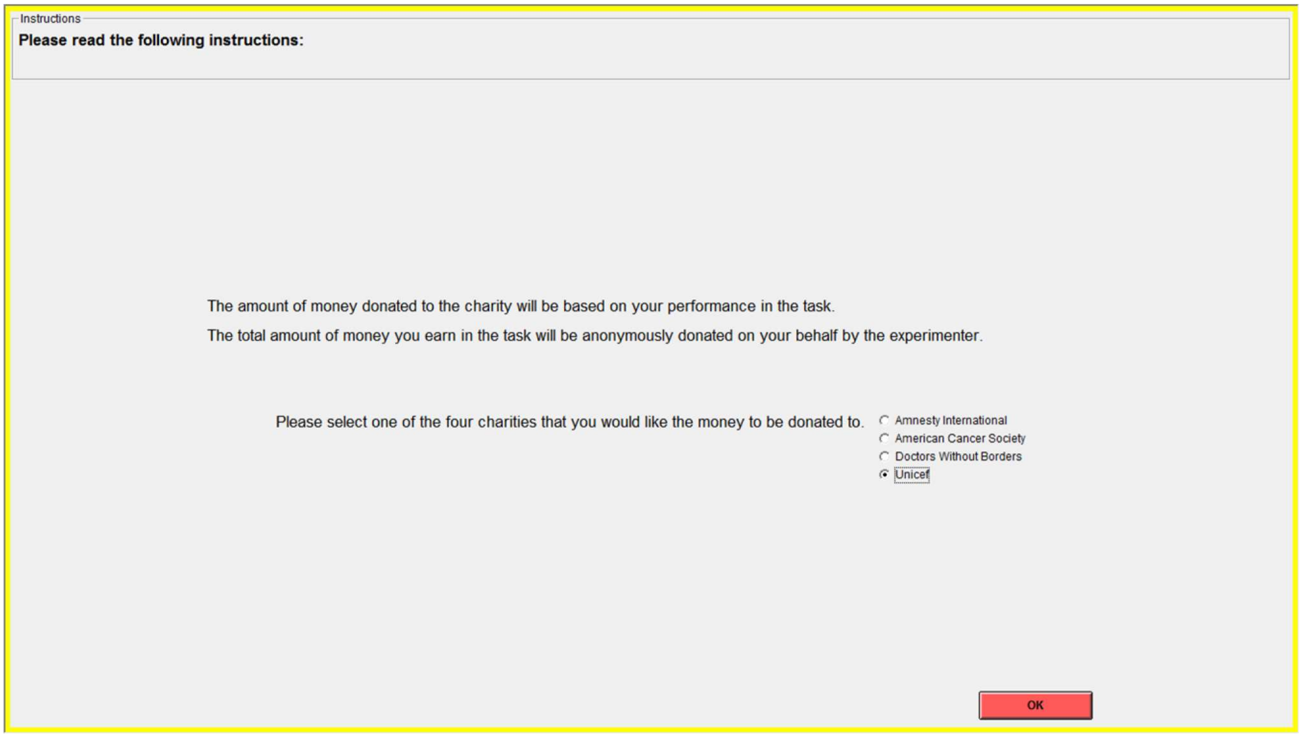

Fig I: Experiment Instructions—Charity Choice

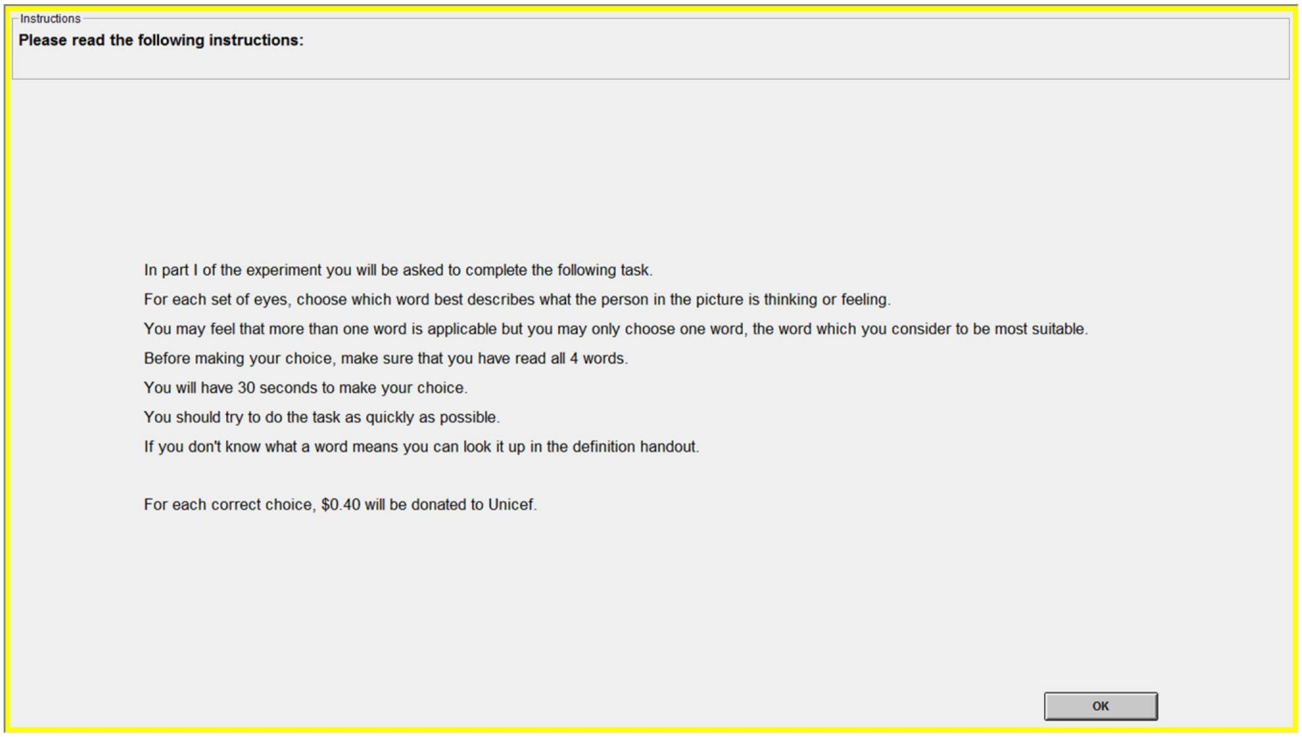

Fig J: Experiment Instructions—Charity Condition, Continued

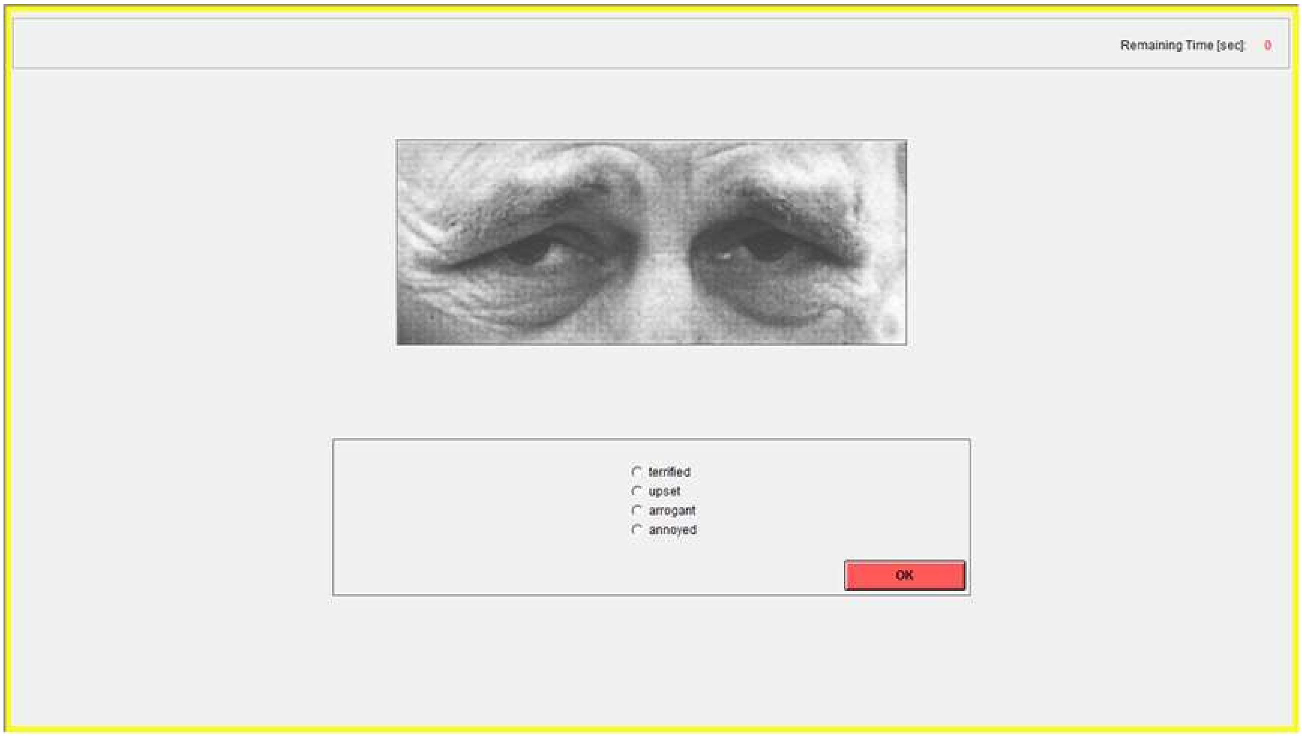

Fig K: Example of RMET Decision Screen

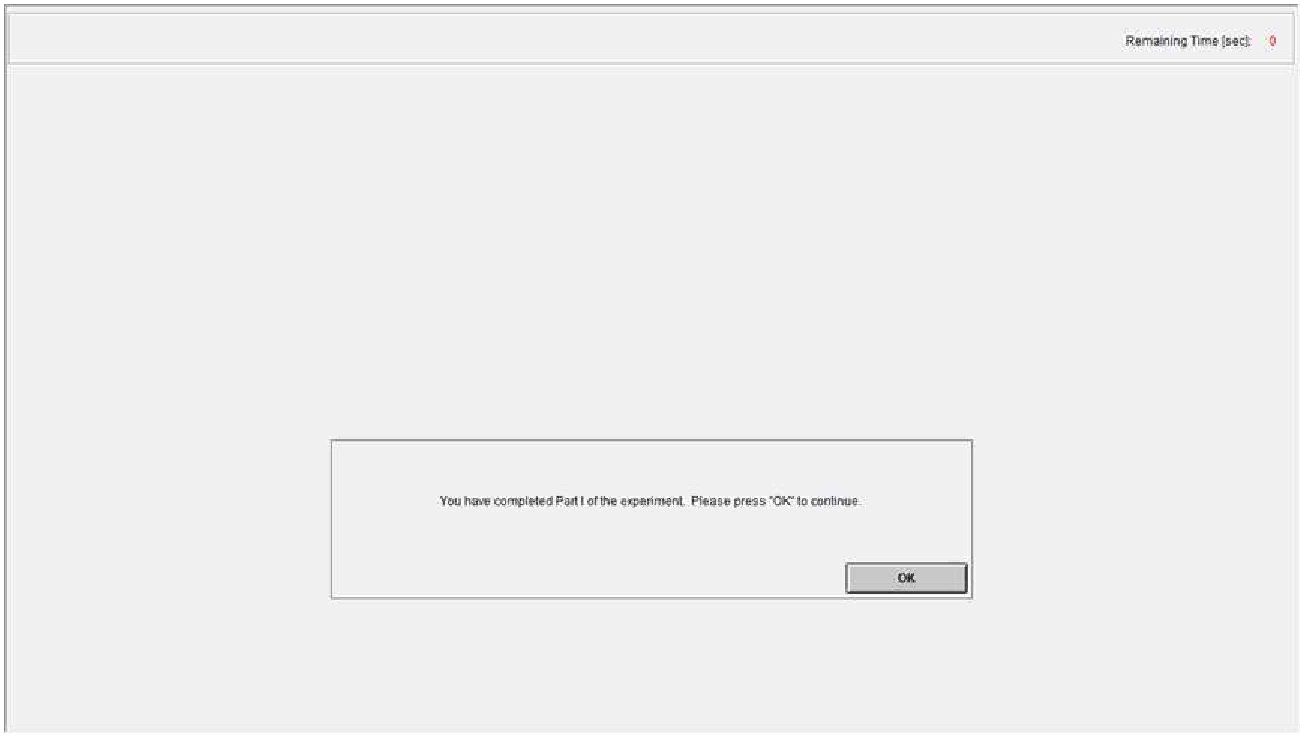

Fig L: Results Screen After Finishing RMET

4 Reading the Mind in the Eyes Test

In this section, we give detailed information on the Reading the Mind in the Eyes Test (RMET) given to subjects in the experiment. We use the revised version of the RMET [12] which consists of 36 pictures of the area around a person's eyes. Each subject was asked to select one of four words that best described what the person is thinking or feeling (for precise instructions please see Fig B, Fig C, Fig E, and Fig J above). In section 4.1, we present the questions from the RMET in the order displayed to the subjects. Each subject saw one picture and set of four words at a time. In section 4.2, we give screenshots of the word definition handouts that each subject received prior to the RMET task. Finally, in Section 4.3, we give the answer for each RMET question.

4.1 Questions

(Practice)

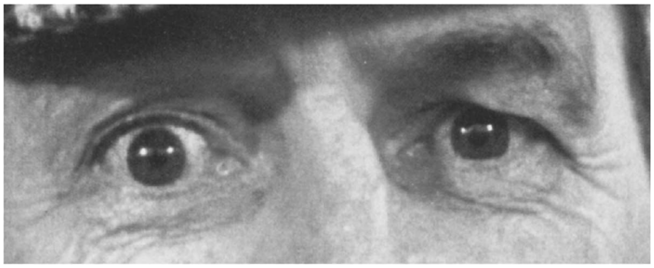

Jealous
Panicked
Arrogant
Hateful

(1)

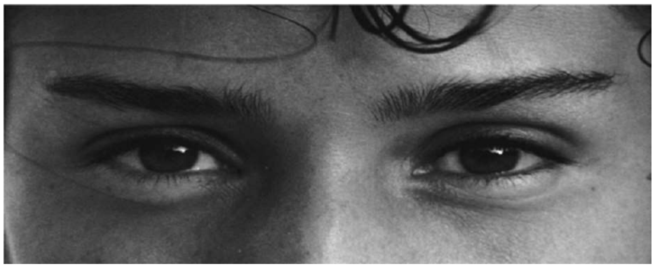

Playful
Comforting
Irritated
Bored

(2)

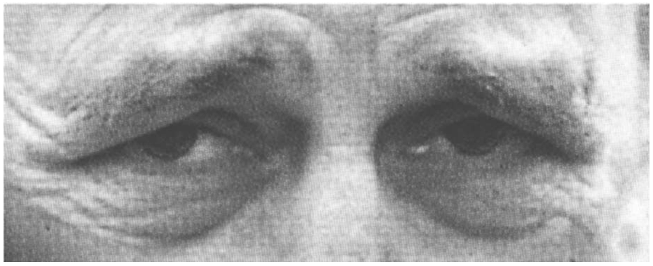

Terrified
Upset
Arrogant
Annoyed

(3)

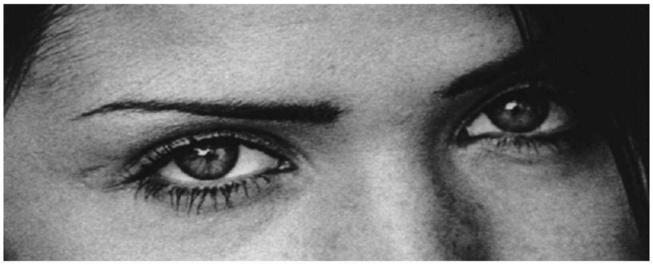

Joking
Flustered
Desire
Convinced

(4)

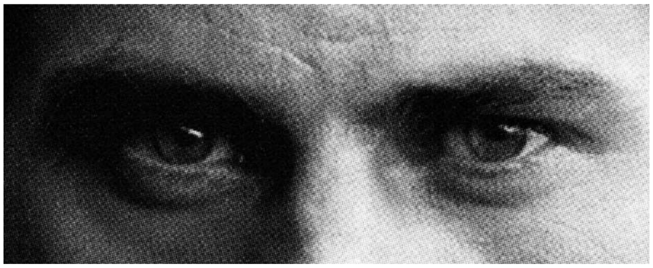

Joking
Insisting
Amused
Relaxed

(5)

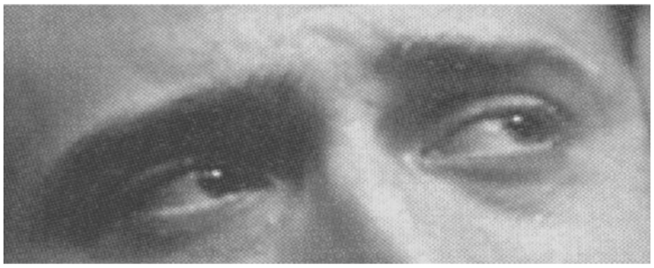

Irritated
Sarcastic
Worried
Friendly

(6)

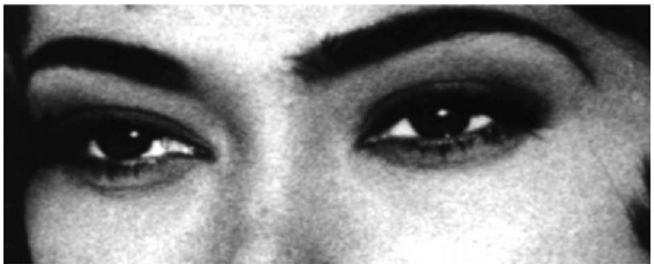

Aghast
Fantasizing
Impatient
Alarmed

(7)

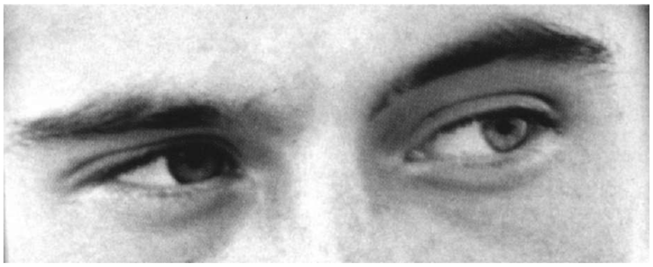

Apologetic
Friendly
Uneasy
Dispirited

(8)

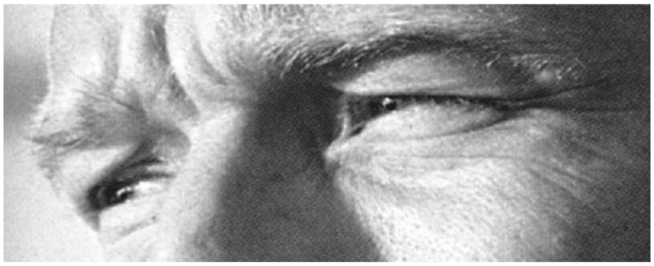

Despondent
Relieved
Shy
Excited

(9)

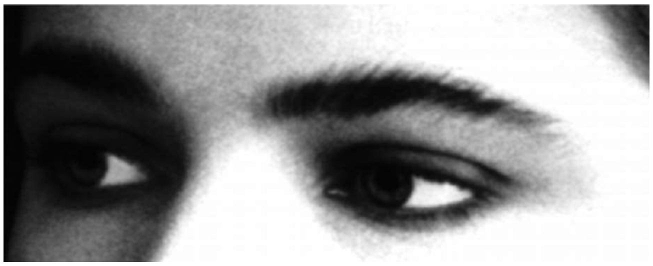

Annoyed
Hostile
Horrified
Preoccupied

(10)

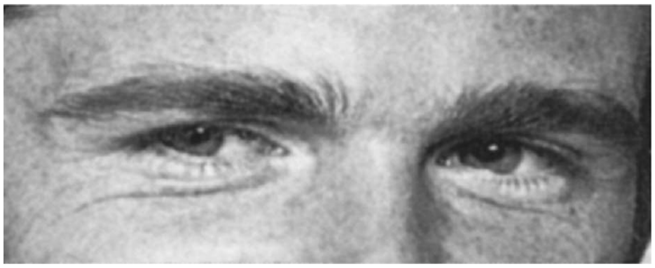

Cautious
Insisting
Bored
Aghast

(11)

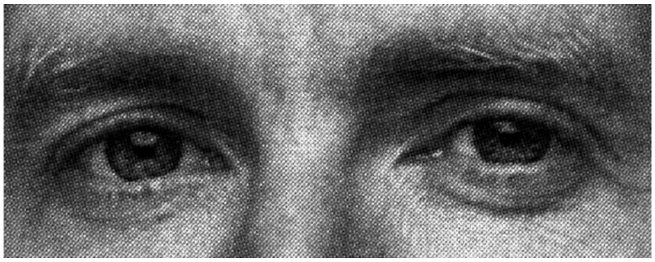

Terrified
Amused
Regretful
Flirtatious

(12)

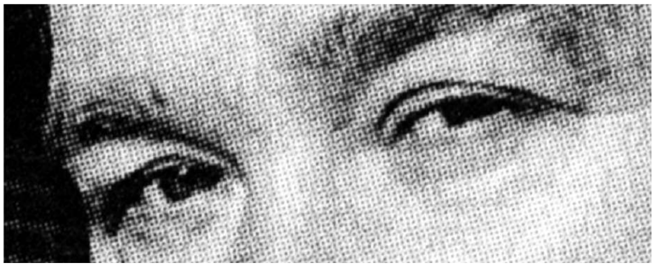

Indifferent
Embarrassed
Skeptical
Dispirited

(13)

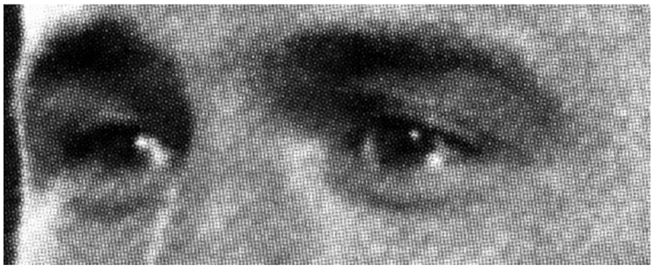

Decisive
Anticipating
Threatening
Shy

(14)

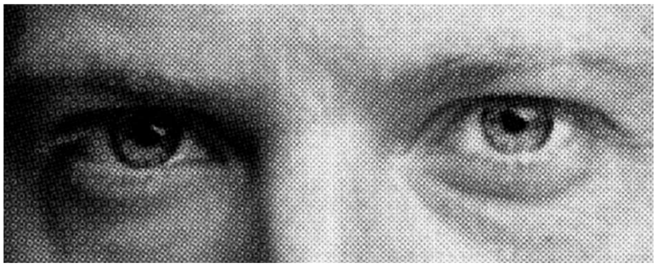

Irritated
Disappointed
Depressed
Accusing

(15)

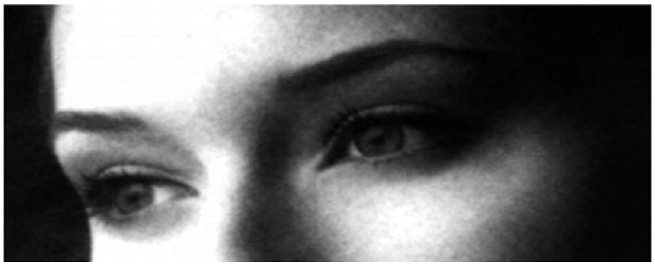

Contemplative
Flustered
Encouraging
Amused

(16)

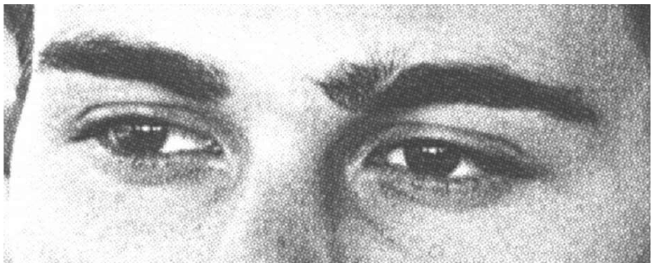

Irritated
Thoughtful
Encouraging
Sympathetic

(17)

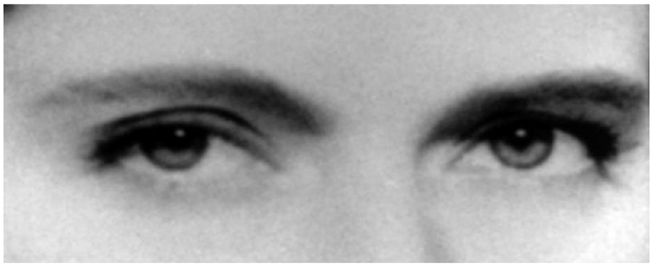

Doubtful
Affectionate
Playful
Aghast

(18)

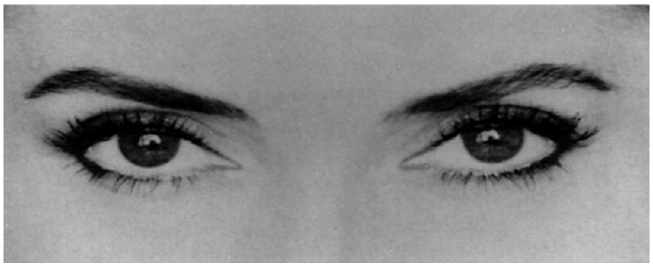

Decisive
Amused
Aghast
Bored

(19)

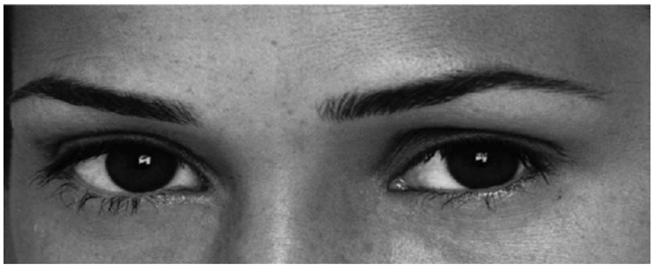

Arrogant
Grateful
Sarcastic
Tentative

(20)

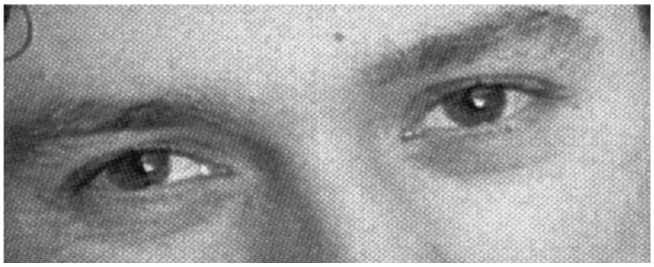

Dominant
Friendly
Guilty
Horrified

(21)

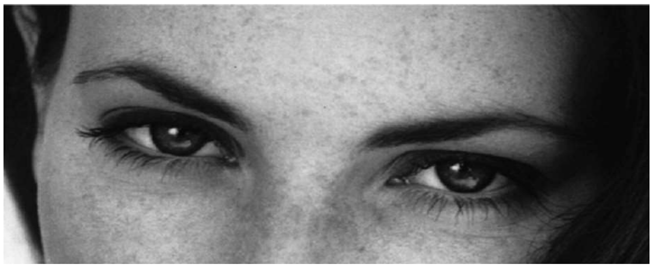

Embarrassed
Fantasizing
Confused
Panicked

(22)

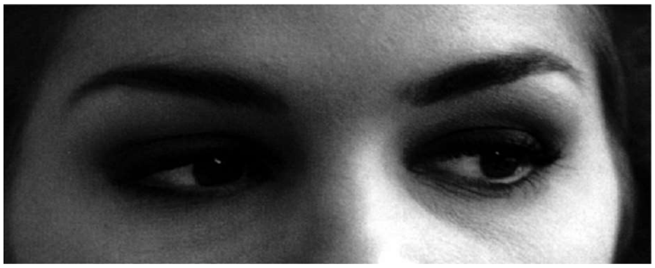

Preoccupied
Grateful
Insisting
Imploring

(23)

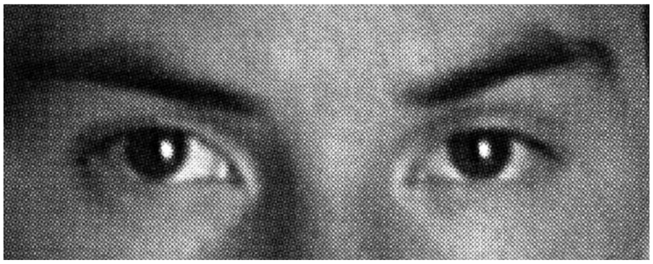

Contented
Apologetic
Defiant
Curious

(24)

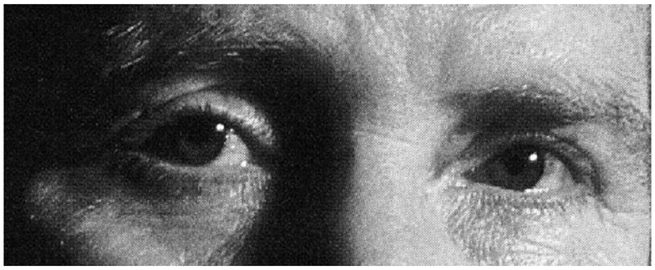

Pensive
Irritated
Exited
Hostile

(25)

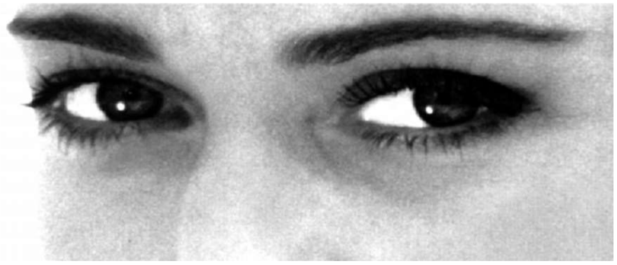

Panicked
Incredulous
Despondent
Interested

(26)

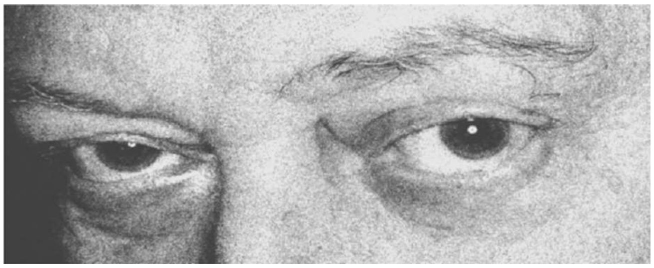

Alarmed
Shy
Hostile
Anxious

(27)

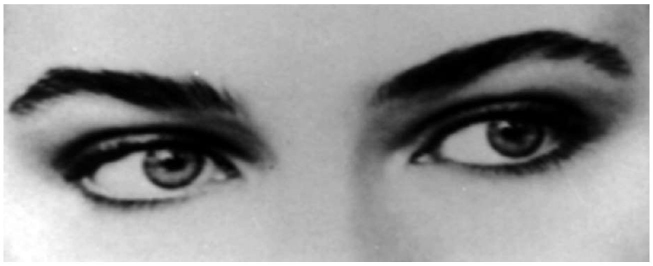

Joking
Cautious
Arrogant
Reassuring

(28)

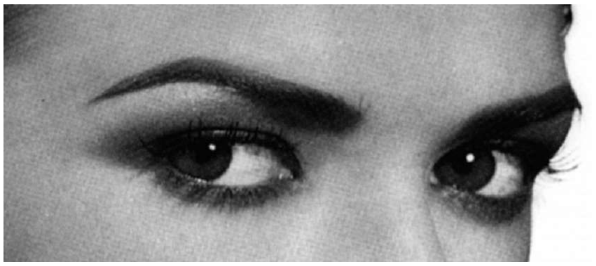

Interested
Joking
Affectionate
Contented

(29)

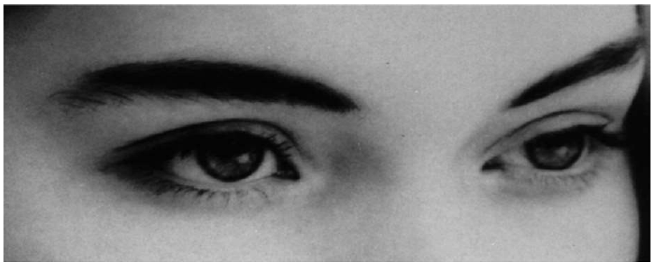

Impatient
Aghast
Irritated
Reflective

(29) 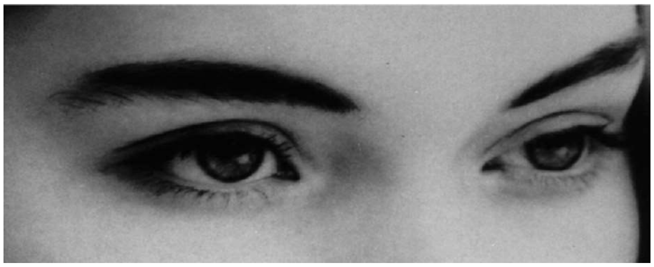

Impatient
Aghast
Irritated
Reflective

(30) 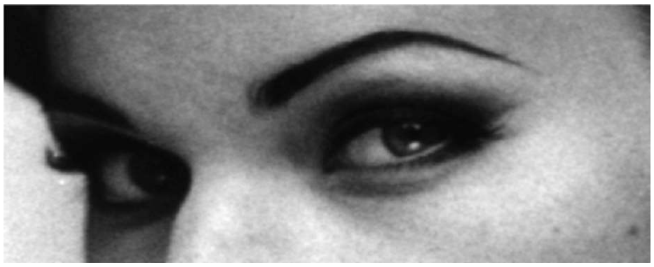

Grateful
Flirtatious
Hostile
Disappointed

(31) 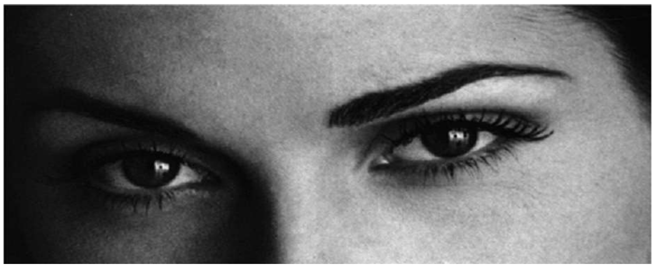

Ashamed
Confident
Joking
Dispirited

(32) 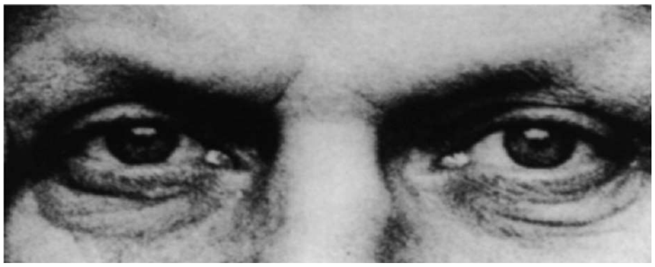

Serious
Ashamed
Bewildered
Alarmed

(33) 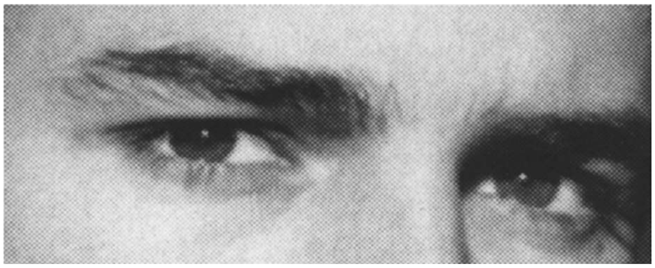

Embarrassed
Guilty
Fantasizing
Concerned

(34)

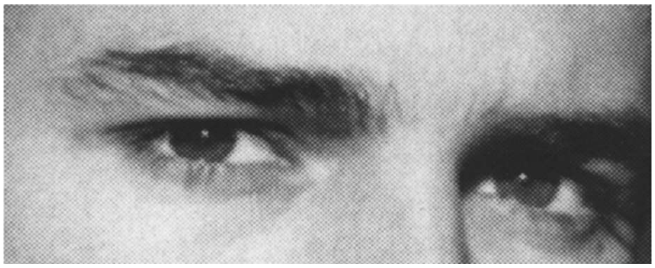

Aghast
Baffled
Distrustful
Terrified

(35)

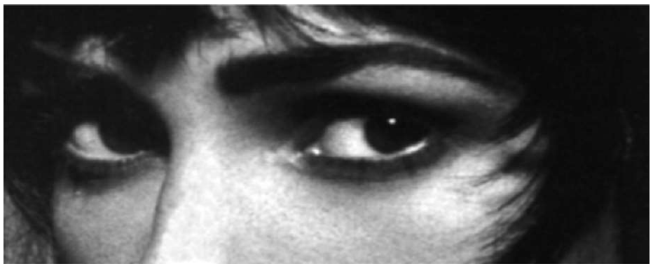

Puzzled
Nervous
Insisting
Contemplative

(36)

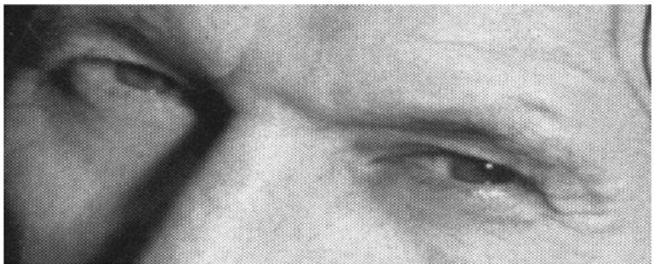

Ashamed
Nervous
Suspicious
Indecisive

4.2 Definitions

WORD DEFINITIONS

Word	Definition	Example
ACCUSING	blaming	The policeman was accusing the man of stealing a wallet.
AFFECTIONATE	showing fondness towards someone	Most mothers are affectionate to their babies by giving them lots of kisses and cuddles.
AGHAST	horrified, astonished, alarmed	Jane was aghast when she discovered her house had been burgled.
ALARMED	fearful, worried, filled with anxiety	Claire was alarmed when she thought she was being followed home.
AMUSED	finding something funny	I was amused by a funny joke someone told me.
ANNOYED	irritated, displeased	Jack was annoyed when he found out he had missed the last bus home.
ANTICIPATING	expecting	At the start of the football match, the fans were anticipating a quick goal.
ANXIOUS	worried, tense, uneasy	The student was feeling anxious before taking her final exams.
APOLOGETIC	feeling sorry	The waiter was very apologetic when he spilt soup all over the customer.
ARROGANT	conceited, self-important, having a big opinion of oneself	The arrogant man thought he knew more about politics than everyone else in the room.
ASHAMED	overcome with shame or guilt	The boy felt ashamed when his mother discovered him stealing money from her purse.
ASSERTIVE	confident, dominant, sure of oneself	The assertive woman demanded that the shop give her a refund.
BAFFLED	confused, puzzled, dumbfounded	The detectives were completely baffled by the murder case.
BEWILDERED	utterly confused, puzzled, dazed	The child was bewildered when visiting the big city for the first time.
CAUTIOUS	careful, wary	Sarah was always a bit cautious when talking to someone she did not know.
COMFORTING	consoling, compassionate	The nurse was comforting the wounded soldier.
CONCERNED	worried, troubled	The doctor was concerned when his patient took a turn for the worse.
CONFIDENT	self-assured, believing in oneself	The tennis player was feeling very confident about winning his match.
CONFUSED	puzzled, perplexed	Lizzie was so confused by the directions given to her, she got lost.
CONTEMPLATIVE	reflective, thoughtful, considering	John was in a contemplative mood on the eve of his 60th birthday.
CONTENTED	satisfied	After a nice walk and a good meal, David felt very contented.
CONVINCED	certain, absolutely positive	Richard was convinced he had come to the right decision.
CURIOUS	inquisitive, inquiring, prying	Louise was curious about the strange shaped parcel.
DECIDING	making your mind up	The man was deciding whom to vote for in the election.
DECISIVE	already made your mind up	Jane looked very decisive as she walked into the polling station.

Fig M: Word Definitions—Page 1

DEFIANT insolent, bold, don't care what anyone else thinks

The animal protester remained defiant even after being sent to prison.

DEPRESSED miserable

George was depressed when he didn't receive any birthday cards.

DESIRE passion, lust, longing for

Kate had a strong desire for chocolate.

DESPONDENT gloomy, despairing, without hope

Gary was despondent when he did not get the job he wanted.

DISAPPOINTED displeased, disgruntled

Manchester United fans were disappointed not to win the Championship.

DISPIRITED glum, miserable, low

Adam was dispirited when he failed his exams.

DISTRUSTFUL suspicious, doubtful, wary

The old woman was distrustful of the stranger at her door.

DOMINANT commanding, bossy

The sergeant major looked dominant as he inspected the new recruits.

DOUBTFUL dubious, suspicious, not really believing

Mary was doubtful that her son was telling the truth.

DUBIOUS doubtful, suspicious

Peter was dubious when offered a surprisingly cheap television in a pub.

EAGER keen

On Christmas morning, the children were eager to open their presents.

EARNEST having a serious intention

Harry was very earnest about his religious beliefs.

EMBARRASSED ashamed

After forgetting a colleague's name, Jenny felt very embarrassed.

ENCOURAGING hopeful, heartening, supporting

All the parents were encouraging their children in the school sports day.

ENTERTAINED absorbed and amused or pleased by something

I was very entertained by the magician.

ENTHUSIASTIC very eager, keen

Susan felt very enthusiastic about her new fitness plan.

FANTASIZING daydreaming

Emma was fantasizing about being a film star.

FASCINATED captivated, really interested

At the seaside, the children were fascinated by the creatures in the rock pools.

FEARFUL terrified, worried

In the dark streets, the women felt fearful.

FLIRTATIOUS brazen, saucy, teasing, playful

Connie was accused of being flirtatious when she winked at a stranger at a party.

FLUSTERED confused, nervous and upset

Sarah felt a bit flustered when she realized how late she was for the meeting and that she had forgotten an important document.

FRIENDLY sociable, amiable

The friendly girl showed the tourists the way to the town Centre.

GRATEFUL thankful

Kelly was very grateful for the kindness shown by the stranger.

GUILTY feeling sorry for doing something wrong

Charlie felt guilty about having an affair.

HATEFUL showing intense dislike

The two sisters were hateful to each other and always fighting.

HOPEFUL optimistic

Larry was hopeful that the post would bring good news.

Fig N: Word Definitions—Page 2

HORRIFIED	terrified, appalled<br>The man was horrified to discover that his new wife was already married.	JEALOUS	envious<br>Tony was jealous of all the taller, better-looking boys in his class.
HOSTILE	unfriendly<br>The two neighbors were hostile towards each other because of an argument about loud music.	JOKING	being funny, playful<br>Gary was always joking with his friends.
IMPATIENT	restless, wanting something to happen soon<br>Jane grew increasingly impatient as she waited for her friend who was already 20 minutes late.	NERVOUS	apprehensive, tense, worried<br>Just before her job interview, Alice felt very nervous.
IMPLORING	begging, pleading<br>Nicola looked imploring as she tried to persuade her dad to lend her the car.	OFFENDED	insulted, wounded, having hurt feelings<br>When someone made a joke about her weight, Martha felt very offended.
INCREDULOUS	not believing<br>Simon was incredulous when he heard that he had won the lottery.	PANICKED	distraught, feeling of terror or anxiety<br>On waking to find the house on fire, the whole family was panicked.
INDECISIVE	unsure, hesitant, unable to make your mind up<br>Tammy was so indecisive that she couldn't even decide what to have for lunch.	PENSIVE	thinking about something slightly worrying<br>Susie looked pensive on the way to meeting her boyfriend's parents for the first time.
INDIFFERENT	disinterested, unresponsive, don't care<br>Terry was completely indifferent as to whether they went to the cinema or the pub.	PERPLEXED	bewildered, puzzled, confused<br>Frank was perplexed by the disappearance of his garden gnomes.
INSISTING	demanding, persisting, maintaining<br>After a work outing, Frank was insisting he paid the bill for everyone.	PLAYFUL	full of high spirits and fun<br>Neil was feeling playful at his birthday party.
INSULTING	rude, offensive<br>The football crowd was insulting the referee after he gave a penalty.	PREOCCUPIED	absorbed, engrossed in one's own thoughts<br>Worrying about her mother's illness made Debbie preoccupied at work
INTERESTED	inquiring, curious<br>After seeing Jurassic Park, Hugh grew very interested in dinosaurs.	PUZZLED	perplexed, bewildered, confused<br>After doing the crossword for an hour, June was still puzzled by one clue.
INTRIGUED	very curious, very interested<br>A mystery phone call intrigued Zoe.	REASSURING	supporting, encouraging, giving someone confidence<br>Andy tried to look reassuring as he told his wife that her new dress did suit her.
IRRITATED	exasperated, annoyed<br>Frances was irritated by all the junk mail she received.	REFLECTIVE	contemplative, thoughtful<br>George was in a reflective mood as he thought about what he'd done with his life.
		REGRETFUL	sorry<br>Lee was always regretful that he had never travelled when he was younger.

Fig O: Word Definitions—Page 3

RELAXED	taking it easy, calm, carefree<br>On holiday, Pam felt happy and relaxed.
RELIEVED	freed from worry or anxiety<br>At the restaurant, Ray was relieved to find that he had not forgotten his wallet.
RESENTFUL	bitter, hostile<br>The businessman felt very resentful towards his younger colleague who had been promoted above him.
SARCASTIC	cynical, mocking, scornful<br>The comedian made a sarcastic comment when someone came into the theatre late.
SATISFIED	content, fulfilled<br>Steve felt very satisfied after he had got his new flat just how he wanted it.
SKEPTICAL	doubtful, suspicious, mistrusting<br>Patrick looked skeptical as someone read out his horoscope to him.
SERIOUS	solemn, grave<br>The bank manager looked serious as he refused Nigel an overdraft.
STERN	severe, strict, firm<br>The teacher looked very stern as he told the class off.
SUSPICIOUS	disbelieving, suspecting, doubting<br>After Sam had lost his wallet for the second time at work, he grew suspicious of one of his colleagues.
SYMPATHETIC	kind, compassionate<br>The nurse looked sympathetic as she told the patient the bad news.
TENTATIVE	hesitant, uncertain, cautious<br>Andrew felt a bit tentative as he went into the room full of strangers.
TERRIFIED	alarmed, fearful<br>The boy was terrified when he thought he saw a ghost.
THOUGHTFUL	thinking about something<br>Phil looked thoughtful as he sat waiting for the girlfriend he was about to finish with.
THREATENING	menacing, intimidating<br>The large, drunken man was acting in a very threatening way.
UNEASY	unsettled, apprehensive, troubled<br>Karen felt slightly uneasy about accepting a lift from the man she had only met that day.
UPSET	agitated, worried, uneasy<br>The man was very upset when his mother died.
WORRIED	anxious, fretful, troubled<br>When her cat went missing, the girl was very worried.

Fig P: Word Definitions—Page 4

4.3 Answers

Table A: RMET Answers

Question	Answer	Question	Answer
(1)	Playful	(19)	Tentative
(2)	Upset	(20)	Friendly
(3)	Desire	(21)	Fantasizing
(4)	Insisting	(22)	Preoccupied
(5)	Worried	(23)	Defiant
(6)	Fantasizing	(24)	Pensive
(7)	Uneasy	(25)	Interested
(8)	Despondent	(26)	Hostile
(9)	Preoccupied	(27)	Cautious
(10)	Cautious	(28)	Interested
(11)	Regretful	(29)	Reflective
(12)	Skeptical	(30)	Flirtatious
(13)	Anticipating	(31)	Confident
(14)	Accusing	(32)	Serious
(15)	Contemplative	(33)	Concerned
(16)	Thoughtful	(34)	Distrustful
(17)	Doubtful	(35)	Nervous
(18)	Decisive	(36)	Suspicious

Although not included in the analysis the answer for the practice question is Panicked.

5 Descriptive Statistics and Hypothesis Tests

Fig 2, from the main article, presents the histogram of RMET scores by treatment and gender. No significant gender difference was found between the variances in the Baseline (Variance ratio test , f=1.26, p=0.52) and Individual (Variance ratio test, f=1.40, p=0.38) conditions. In the Winner-take-all condition, the variance for females is statistically larger than the variance for males at the 10% level (Variance ratio test, f=0.53, p=0.09). In the Charity condition, the variance for male RMET scores was significantly larger than the variance for females (Variance ratio test, f=3.56, p=0.00).

For each variable used in the data analysis, Table B provides a description and how

Table B: Description of Variables

	Overall
RMET Score	Sum of correct answers in RMET test out of 36.
Correct Answer	Dummy variable equal to one if subject answered RMET question correctly.
Female	Dummy variable equal to one if female.
Male	Dummy variable equal to one if male.
Age	Equal to the age of subject.
Number of Years Living in U.S.	Equal to the number of years lived in the U.S.
Native English Speaker	Equal to one if the subject is a native English speaker.
Cognitive Reflection Test	Equal to the sum of correct answers in the Cognitive Reflection Test.
Number of Economics Classes	Equal to the number of economics courses taken.
Number of Statistics Classes	Equal to the number of statistics courses taken.
Average Question Time	Equal to the average time taken for each question by a given subject.
Individual	Dummy variable equal to one if subject was in the Individual condition.
Winner-take-all	Dummy variable equal to one if subject was in the Winner-take-all condition.
Charity	Dummy variable equal to one if subject was in the Charity condition.
Individual x female	Dummy variable equal to one if subject was in the Individual condition and female.
Individual x male	Dummy variable equal to one if subject was in the Individual condition and male.
Winner-take-all x female	Dummy variable equal to one if subject was in the Winner-take-all condition and female.
Winner-take-all x male	Dummy variable equal to one if subject was in the Winner-take-all condition and male.
Charity x female	Dummy variable equal to one if subject was in the Charity condition and female.
Charity x male	Dummy variable equal to one if subject was in the Charity condition and male.

Table C: Summary Statistics

	Overall	Baseline	Individual	Winner-take-all	Charity
RMET Score	27.45	27.61	27.31	27.09	27.87
Female (Count)	144	41	40	37	26
Male (Count)	94	23	18	27	26
Age	20.13	19.77	20.09	20.19	20.56
Age (Minimum)	18	18	18	18	28
Age (Max)	30	23	28	28	30
Number of Years Living in U.S.	16.74	16.41	17.62	15.58	17.62
Native English Speaker	0.48	0.42	0.62	0.45	0.44
Cognitive Reflection Test	1.09	1.11	1.12	1.11	1.00
Number of Economics Classes	1.32	1.63	1.21	0.97	1.50
Number of Statistics Classes	1.04	1.06	1.03	0.89	1.19
Average Question Time	12.85	12.06	12.67	12.96	13.87
Money Earned from RMET	7.79	0	10.92	10.00	11.15*
Money Donated to Charity	2.44*	0	0	0	11.15*
Take Home Pay	12.35	7	17.92	17	7
Observations	238	64	58	64	52

All statistics are averages unless otherwise noted. *Money was donated to subjects selected charity.

the variable was coded. Table C gives the summary statistics of the variables used in both the main paper and the supplementary appendix. On average 61% of subjects were female and the average age was approximately 20 years old. Scores on the cognitive reflection test were coded to be equal to the sum of the correct answers. The Cronbach alpha for the three questions on the cognitive reflection test was 0.70. Overall, subjects received a $7 show up payment, and on average earned $7.79 on the RMET task. The average time spent by subjects across the treatments was similar. Compared to the Baseline no significance difference in average time spent on the questions was found for the Individual condition (Wilcoxon rank sum test, z=-1.164, p=0.2445) or the Winner-take-all condition (Wilcoxon rank sum test, z=-1.425, p=0.1542). Subjects took longer on average in the Charity condition compared to the Baseline, this difference is statistically significant (Wilcoxon rank sum test, z=3.026, p=0.003).

The average time that subjects took to answer questions is negatively correlated with RMET scores. As a result, it is important to understand if the gender difference we observe

in the treatments is driven by changes in the length of time individuals take to answer each question. Table D presents regression results examining how both the treatments and gender influenced the average time taken for a given RMET question. The first column shows that the Charity condition was associated with a statistically significant increase in the time taken, but neither the Individual nor Winner-take-all conditions statistically differed from the Baseline condition. Column 2 interacts the conditions with a dummy variable for female. It appears, that females took significantly longer on the Charity condition relative to the Baseline but no significant interaction was found in the Individual or Winner-take-all conditions. As a result, it appears that changes in the time taken are unable to explain the gender differences that occur in the Individual and Winner-take-all conditions.

In the main paper, Fig 1 (A) presents the pooled results across the different conditions. No significant difference in RMET scores occurs between the Baseline condition and the Individual condition (Wilcoxon rank sum test, z=-0.773, p=0.440). Similarly, no significant difference was found in the Winner-take-all condition (Wilcoxon rank sum test, z=-0.469, p=0.639) or the Charity condition (Wilcoxon rank sum test, z=0.385, p=0.700) relative to the Baseline condition. For Fig 1 (B) in the main paper, there was a statistically significant decrease in RMET scores for females in the Individual condition (Wilcoxon rank sum, z=-1.814, p=0.070) and the Winner-take-all condition (Wilcoxon rank sum, z=-1.894, p=0.058) relative to the Baseline condition. No significant difference for females occurred in the Charity condition compared to the Baseline condition (Wilcoxon rank sum test, z=0.136, p=0.892). For male RMET scores, the Individual condition (Wilcoxon rank sum test, z=0.912, p=0.362) and the Charity condition (Wilcoxon rank sum test, z=0.836, p=0.403) are not significantly different compared to the Baseline condition. In the Winner-take-all condition male RMET scores were higher on average compared to the Baseline condition, this difference is significant at the 11% level (Wilcoxon rank sum test, z=1.614, p=0.107). These non-parametric tests are preformed on the data not controlling for additional factors that may influence individual RMET. The next section presents regression results that accounts for additional covariates

Table D: Average Question Time Taken on RMET by Treatment and Demographics

	(1)<br>Average Question Time	(2)<br>Average Question Time
Charity	1.75***	0.20
	(0.61)	(1.10)
Individual	0.96	0.33
	(0.59)	(1.10)
Winner-take-all	0.77	-0.60
	(0.71)	(1.20)
Female	-1.02**	-2.54**
	(0.49)	(1.08)
Native English Speaker	-0.85*	-1.03**
	(0.47)	(0.48)
Years living in U.S.	-0.11**	-0.10**
	(0.05)	(0.05)
Cognitive Reflection Test	-0.57***	-0.62***
	(0.21)	(0.21)
Charity x Female		2.65**
		(1.34)
Individual x Female		1.06
		(1.32)
Winner-take-all x Female		2.22
		(1.48)
Intercept	15.46***	16.46***
	(1.09)	(1.33)
N	238	238
R^2	0.123	0.141

Regressions are ordinary least squares (OLS). Clustered standard errors at the subject level are in parentheses
* $p < 0.10$, ** $p < 0.05$, *** $p < 0.01$

that impact RMET giving more precise estimates of the relationship between the experiment conditions and RMET.

6 Regression Analysis and Robustness Checks

In this section, we present more detailed regression analysis from the main paper and present additional robust checks. Table E presents a more detailed analysis of the ordinary least squares regressions in Table 2 from the main paper. Column (3) and column (4) are the regressions in column (A) and (B) from Table 2 in the main paper, but include the coefficients for the control variables. Gender, whether English is the subject's first language, the number of years the subject has lived in the U.S, and cognitive ability are all positive and statistically significant factors in RMET score. In addition, average question time is negatively correlated with RMET score suggesting that people who took longer to answer the question were more likely to choose the incorrect answer. These four variables are listed as controls in the regression results. For column (A), we reject the joint hypothesis that these four variables are equal to zero (F-test, F(4,237)=13.94, p=0.00). Similar results are found for column (B) (F-test, F(4,237)=15.32). Similarly Table F presents the same regressions as Table E but interacts a dummy variable for female with the experimental conditions. These tables are both equivalent, but we presented the regressions in Table E in the main paper for clarity. Table G uses the same regressions as Table E but uses hetereoskedastically robust standard errors. There is essentially no difference in the results between the two error assumptions.

Table H presents random effects probit regressions on the probability that a subject answers a given RMET question correctly. Columns (2) and (4) were used to calculate the change in predicted probability that is presented in columns (C) and (D) of Table 2 in the main paper. The regressions in columns (C) and (D) include control variables, subject specific effects, and question fixed effects. For the control variables in column (2), using a

Table E: Predicting RMET Score Pure Gender Effect by Treatment and Demographics

	(1)<br>RMET Score	(2)<br>RMET Score	(3)<br>RMET Score	(4)<br>RMET Score
Charity	0.26	0.17	0.68	
	(0.65)	(0.61)	(0.57)	
Individual	−0.30	−0.78	−0.57	
	(0.59)	(0.56)	(0.56)	
Winner-take-all	−0.52	−0.39	−0.18	
	(0.70)	(0.63)	(0.61)	
Female		0.81*	1.03**	2.87***
		(0.46)	(0.45)	(0.83)
Native English Speaker		1.32***	0.99**	1.01**
		(0.47)	(0.44)	(0.44)
Years living in U.S.		0.14***	0.13**	0.13***
		(0.05)	(0.05)	(0.05)
Average Question Time			−0.22***	−0.20***
			(0.07)	(0.07)
Cognitive Reflection Test			0.50**	0.53***
			(0.20)	(0.20)
Charity x Female				0.33
				(0.66)
Charity x Male				1.48
				(0.95)
Individual x Female				−1.42**
				(0.66)
Individual x Male				0.95
				(1.04)
Competition x Female				−1.62**
				(0.82)
Competition x Male				2.03**
				(0.91)
Intercept	27.61***	24.18***	26.52***	25.04***
	(0.44)	(0.91)	(1.56)	(1.73)
N	238	238	238	238
R^2	0.006	0.135	0.212	0.248

Regressions are ordinary least squares (OLS). Clustered standard errors at the subject level are in parentheses
* $p < 0.10$, ** $p < 0.05$, *** $p < 0.01$

Table F: Predicting RMET Score with Gender Interactions by Treatment and Demographics

	(1)<br>RMET Score	(2)<br>RMET Score	(3)<br>RMET Score	(4)<br>RMET Score
Charity	0.26	0.17	0.68	1.48
	(0.65)	(0.61)	(0.57)	(0.95)
Individual	-0.30	-0.78	-0.57	0.95
	(0.59)	(0.56)	(0.56)	(1.04)
Winner-take-all	-0.52	-0.39	-0.18	2.03^{**}
	(0.70)	(0.63)	(0.61)	(0.91)
Female		0.81^{*}	1.03^{**}	2.87^{***}
		(0.46)	(0.45)	(0.83)
Native English Speaker		1.32^{***}	0.99^{**}	1.01^{**}
		(0.47)	(0.44)	(0.44)
Years living in U.S.		0.14^{***}	0.13^{**}	0.13^{***}
		(0.05)	(0.05)	(0.05)
Average Question Time			-0.22^{***}	-0.20^{***}
			(0.07)	(0.07)
Cognitive Reflection Test			0.50^{**}	0.53^{***}
			(0.20)	(0.20)
Charity x Female				-1.15
				(1.16)
Individual x Female				-2.37^{*}
				(1.23)
Winner-take-all x Female				-3.65^{***}
				(1.26)
Intercept	27.61^{***}	24.18^{***}	26.52^{***}	25.04^{***}
	(0.44)	(0.91)	(1.56)	(1.73)
N	238	238	238	238

Regressions are ordinary least squares (OLS). Clusterd standard errors at the subject level are in parentheses
* $p < 0.10$, ** $p < 0.05$, *** $p < 0.01$

Table G: Predicting RMET Score by Treatment and Demographics with Robust Standard Errors

	(1) RMET Score	(2) RMET Score	(3) RMET Score	(4) RMET Score
Charity	0.26	0.17	0.68	
	(0.65)	(0.61)	(0.57)	
Individual	−0.30	−0.78	−0.57	
	(0.59)	(0.56)	(0.56)	
Winner-take-all	−0.52	−0.39	−0.18	
	(0.70)	(0.63)	(0.61)	
Female		0.81*	1.03**	2.87***
		(0.46)	(0.45)	(0.83)
Native English Speaker		1.32***	0.99**	1.01**
		(0.47)	(0.44)	(0.44)
Years living in U.S.		0.14***	0.13**	0.13***
		(0.05)	(0.05)	(0.05)
Average Question Time			−0.22***	−0.20***
			(0.07)	(0.07)
Cognitive Reflection Test			0.50**	0.53***
			(0.20)	(0.20)
Charity x Female				0.33
				(0.66)
Charity x Male				1.48
				(0.95)
Individual x Female				−1.42**
				(0.66)
Individual x Male				0.95
				(1.04)
Winner-take-all x Female				−1.62**
				(0.82)
Winner-take-all x Male				2.03**
				(0.91)
Intercept	27.61***	24.18***	26.52***	25.04***
	(0.44)	(0.91)	(1.56)	(1.73)
N	238	238	238	238
R^2	0.006	0.135	0.212	0.248

Regressions are ordinary least squares (OLS). Robust standard errors in parentheses

* $p < 0.10$, ** $p < 0.05$, *** $p < 0.01$

chi-squared test we reject the joint hypothesis that the control variables for Native English Speaker, Average Question Time, Cognitive Reflection Test, and Number of Years Lived in the U.S. are jointly equal to zero (Chi-squared Test, χ^4=59.92, p=0.00). Similar results are found for column (4) (Chi-squared Test, χ^4=60.97, p=0.00). The subject specific effect is a random factor and we reject that this factor is equal to zero for column (2) (Chi-squared Test, χ^1=44.36, p=0.00) and column (4) (Chi-squared Test, χ^1=36.66, p=0.00). The question fixed effects allow us to control for any specific questions that may influence the probability of a subject answering a question correctly. We can reject the hypothesis that the coefficients for the question dummy variables are jointly equal to zero for column (2) (Chi-squared Test, χ^{31}=638.72, p=0.00) and column (4) (Chi-squared Test, χ^{31}=638.56, p=0.00).

There is issue in interpretation of the interaction terms in non-linear models when interested in marginal effects [80, 81]. To deal with this issue, we followed ([81] and exploited the fact that our interaction terms were dummy variables. Using the regression results from columns (2) and (4), we calculated the change in predicted probability for each case relative to the Baseline condition, and used the delta method to calculate the standard errors. The results are presented in Table I.

Table H: Predicting Probability of Correct Answer in RMET: Treatment and Gender Interactions

	(1) Correct Answer	(2) Correct Answer	(3) Correct Answer	(4) Correct Answer
Charity	0.02	0.07	0.14*	0.15*
	(0.06)	(0.06)	(0.08)	(0.09)
Individual	−0.03	−0.06	0.08	0.09
	(0.06)	(0.06)	(0.09)	(0.10)
Winner-take-all	−0.04	−0.01	0.18**	0.20**
	(0.06)	(0.06)	(0.08)	(0.09)
Female		0.10**	0.26***	0.29***
		(0.05)	(0.08)	(0.09)
Native English Speaker		0.11**	0.09**	0.11**
		(0.05)	(0.04)	(0.05)
Years living in U.S.		0.01***	0.01***	0.01***
		(0.00)	(0.00)	(0.00)
Average Question Time		−0.02***	−0.02***	−0.02***
		(0.01)	(0.01)	(0.01)
Cognitive Reflection Test		0.05***	0.05***	0.05***
		(0.02)	(0.02)	(0.02)
Individual X Female			−0.21*	−0.24**
			(0.11)	(0.12)
Charity X Female			−0.11	−0.12
			(0.11)	(0.12)
Winner-take-all X Female			−0.32***	−0.35***
			(0.10)	(0.11)
Intercept	0.75***	−0.22	0.52***	−0.37**
	(0.04)	(0.15)	(0.12)	(0.16)
Individual Question Fixed Effects	No	Yes	No	Yes
N	8568	8568	8568	8568
ρ	0.05	0.04	0.03	0.04
χ^2	1.45	745.21	77.10	754.90

Regressions are random effects probit with standard errors in parentheses.

* $p < 0.10$, ** $p < 0.05$, *** $p < 0.01$

Table I: Change in Predicted Probability of Correct Answer in RMET Relative to Baseline

	Individual		Winner-take-all		Charity	
	Change in Predicted Probability	Standard Error	Change in Predicted Probability	Standard Error	Change in Predicted Probability	Standard Error
Female	-0.04^{**}	(0.02)	-0.04^{**}	(0.02)	0.01	(0.02)
	95% CI [-0.08,-0.00]		95% CI [-0.08,-0.00]		95% CI [-0.04,0.05]	
Male	-0.03	(0.03)	0.06^{**}	(0.02)	0.04^{*}	(0.02)
	95% CI [-0.03,0.08]		95% CI [0.01,0.10]		95% CI [-0.01,0.09]	

Predicted probabilities are derived from the random effects probit regression from Table S6 column 4.
Standard errors are calculated using the delta method and are reported in parentheses.
Confidence intervals at the 95% level are reported in brackets.
* $p < 0.10$, ** $p < 0.05$, *** $p < 0.01$

References

[79] Fischbacher U. z-Tree: Zurich toolbox for ready-made economic experiments. Exp. Econ. 2007; 19: 171-178.

[80] Ai C, Norton EC. Interaction terms in logit and probit models. Econ. Lett. 2003; 80: 123-129.

[81] Buis ML. Stata tip 87: Interpretation of interactions in nonlinear models. Stata J. 2010; 10: 305-308.
