# Supplementary material for: Money Affects Theory of Mind Differently by Gender
Source: PLoS One. 2015 Dec 3;10(12):e0143973. doi: 10.1371/journal.pone.0143973 (PMC4669079; doi:10.1371/journal.pone.0143973)
Supplement: S1 Readme — (PDF) [file pone.0143973.s004.pdf]

This file contains the data set used to produce the results in the paper "Money Affects Theory of Mind Differently by Gender." For convenience, the data is provided in two formats. An excel file named “S1 Dataset.xls” and a Stata file (version 14) “S2 Dataset.dta”. Explanations for each variable in the data sets are provided below for reference.

Codebook

1. Subject- A number representing a single subject.
2. Question- The question number from one of the 36 RMET questions.
3. Session- Number equal to one the Session Number.
4. Age- Number equal to the subject’s age.
5. Race- Is the subjects stated ethnicity.
6. Major- Subjects stated college major.
7. Graduate- Whether the student is an undergraduate or graduate student.
8. Econ- Number of economics classes taken previously.
9. Stats- Number of statistics classes taken previously.
10. Political- Subjects selected political view from the following scale: Extremely Liberal, Liberal, Moderate, Conservative, Extremely Conservative.
11. Religion- Subjects stated religion.
12. Birthcountry- Subjects place of birth.
13. English- If yes, English is subjects first language. If no, English is not subjects first language.
14. Usyears- Equal to the number of years the subject has lived in the United States.
15. Crt1- Subject’s answer to CRT question “A bat and a ball cost $1.10 in total. The bat costs $1.00 more than the ball. How much does the ball cost?”
16. Crt2- Subject’s answer to CRT question “If it takes 5 machines 5 minutes to make 5 widgets, how long would it take 100 machines to make 100 widgets?”
17. Crt3- Subject’s answer to CRT question “In a lake, there is a patch of lily pads. Every day, the patch doubles in size. If it takes 48 days for the patch to cover the entire lake, how long would it take for the patch to cover half of the lake?”
18. Tomscore- The number of correct questions on the RMET test.
19. Winner- Equal to one if subject had highest RMET score in their group of four in the Winner-take-all condition. Equal to zero otherwise.
20. Female- Equal to one if the subject was female. Equal to zero if the subject was male.
21. Englishfirst- Equal to one if the subject’s first language is English. Equal to zero otherwise.
22. Cognitive- Equal to the number of CRT questions answered correctly.
23. Incentivized- Equal to one if the subject participated in the Individual condition. Equal to zero otherwise.
24. Charityincentive- Equal to one if the subject participated in the Charity condition. Equal to zero otherwise.
25. Tournament- Equal to one in the subject participated in the Winner-take-all condition. Equal to zero otherwise.
26. Noincentive- Equal to one if the subject participated in the Baseline condition. Equal to zero otherwise.
27. Avgq1-avgq36- Equal to the average correct answer for a given question by treatment condition.

28. Correct- Equal to one if the subject answered the question correctly. Equal to zero otherwise.
29. Incentivizedxfemale- Equal to one if the subject was female and in the Individual condition. Equal to zero otherwise.
30. Tournamentxfemale- Equal to one if the subject was female and in the Winner-take-all condition. Equal to zero otherwise.
31. Charityincentivexfemale- Equal to one if the subject was female and in the Charity condition. Equal to zero otherwise.
32. Q1-q36- Equal to one if the question q1-q36 is equal to the RMET question number in the variable Question. Equal to zero otherwise.
33. Timetaken- The time the subject took to answer the RMET question.
34. Avgtimetaken- The average time taken by a subject to answer an RMET question.
35. Incentivizedxmale- Equal to one if the subject was male and in the Individual condition. Equal to zero otherwise.
36. Tournamentxmale- Equal to one if the subject was male and in the Winner-take-all condition. Equal to zero otherwise.
37. Charityincentivexmale- Equal to one if the subject was male and in the Charity condition. Equal to zero otherwise.
38. Treatcategory- Equal to 1 if Baseline condition, 2 if Individual condition, 3 if Winner-take all condition, and 4 if Charity condition.
39. Avgtime- Equal to the average time taken per question on the RMET by treatment condition.
40. Avgtimefemale- Equal to the average time taken per question on the RMET by treatment condition if subjects were female.
41. Avgtimemale- Equal to the average time taken per question on the RMET by treatment condition if subjects were female.
42. Tomq- Equal to the answer the subjects gave to the RMET question
